# Miniaturized disordered photonic molecule spectrometer

**DOI:** 10.1038/s41377-024-01705-w

**Published:** 2025-03-31

**Authors:** Yujia Zhang, Tom Albrow-Owen, Zhenyu Zhao, Yinpeng Chen, Yaotian Zhao, Hannah Joyce, Tawfique Hasan, Zongyin Yang, Yikai Su, Xuhan Guo

**Affiliations:** 1 State Key Laboratory of Photonics and Communications, School of Information and Electronic Engineering, Shanghai Jiao Tong University, Shanghai, 200240 China; 2 Department of Engineering, University of Cambridge, Cambridge, CB3 0FA UK; 3 College of Information Science and Electronic Engineering, Zhejiang University, Hangzhou, 310027 China

**Keywords:** Applied optics, Other photonics

## Abstract

The burgeoning field of computational spectrometers is rapidly advancing, providing a pathway to highly miniaturized, on-chip systems for in-situ or portable measurements. The performance of these systems is typically limited in its encoder section. The response matrix is largely compromised with redundancies, due to the periodic intensity or overly smooth responses. As such, the inherent interdependence among the physical size, resolution, and bandwidth of spectral encoders poses a challenge to further miniaturization progress. Achieving high spectral resolution necessitates a long optical path length, leading to a larger footprint required for sufficient spectral decorrelation, resulting in a limited detectable free-spectral range (FSR). Here, we report a groundbreaking ultra-miniaturized disordered photonic molecule spectrometer that surpasses the resolution-bandwidth-footprint metric of current spectrometers. This computational spectrometer utilizes complicated electromagnetic coupling to determinately generate quasi-random spectral response matrices, a feature absents in other state-of-the-art systems, fundamentally overcoming limitations present in the current technologies. This configuration yields an effectively infinite FSR while upholding a high Q-factor ( > 7.74 × 10^5^). Through dynamic manipulation of photon frequency, amplitude, and phase, a broad operational bandwidth exceeding 100 nm can be attained with an ultra-high spectral resolution of 8 pm, all encapsulated within an ultra-compact footprint measuring 70 × 50 μm². The disordered photonic molecule spectrometer is constructed on a CMOS-compatible integrated photonics platform, presenting a pioneering approach for high-performance and highly manufacturable miniaturized spectroscopy.

## Introduction

Optical spectroscopy is a critical characterization technique across disciplines employed wherever light can usefully probe matter, across the biological^1–3^ and physical sciences^4^. In recent years, the pursuit of high integration density, low-cost devices have intensified, for use in portable, on-chip or in-situ applications^5^. This has led to the development of a variety of miniaturized spectrometers based on dispersive and interference optics^6–11^. However, in such strategies, inherent interdependency of the physical size, resolution and bandwidth of devices constrains further technological advancement; addressing this three-way trade-off, to produce simultaneously high resolution, high bandwidth and low footprint systems, is a priority within the field. Fourier-transform spectrometers (FTS) rely on optical path differences to generate interferograms and converted to spectral signal via Fourier transform. As the spectral resolution and bandwidth of FTS are determined by the maximum optical path difference and the number of photodetectors according to the Rayleigh criterion, the main challenge is the similar inherent trade-off between spectral performance (operational bandwidth, spectral resolution) and footprint (channel count)^12,13^. The realization of high-performance FTS usually requires a large footprint ranging from several to hundreds of millimeter^14–16^ or watt-scale drive power^17^.

Computational spectrometers are an emerging paradigm of devices that, through software-mediated augmentation, offer a route to circumvent these performance limitations. Harnessing increasingly ubiquitous embedded computing, these systems employ algorithms to deconvolute the interaction of incident light with an ensemble of spectral encoders possessing unique, varied spectral response characteristics, ‘reconstructing’ the original spectrum rather than directly measuring it. Metasurface filter arrays^18,19^ or nanomaterial-based spectral encoders^20–24^ have often been used as the media to generate a spectral response matrix, as well as Photonic-integrated-circuit (PIC)-based systems, employing spectral-to-spatial mapping using dispersive components such as disordered photonic chips^25–28^, stratified waveguide filters^29^, and multimode spiral waveguides^30,31^. On-chip resonators such as microrings are promising candidates because narrow and sharp resonance peaks with high quality-factors (Q-factors) are achievable, leading to sharp transmissions with drastic changes at two closely separated wavelengths, which means high-resolution systems in compact footprints^32^.

However, in all of these cases, the accuracy and stability of the reconstruction are intrinsically dependent on the randomness of the response matrix. Where insufficient diversity is included, redundant information is encoded into the response matrix due to multicollinearity^33^. To this end, minimizing the condition number and optimizing the orthogonality of the response matrix are crucial to increase the reconstruction stability and reliability of numerical solutions. In on-chip resonator-based devices, it is the periodicity of the spectral response which leads to a reduction of the orthogonality. One approach is to incorporate double transmission modes with slightly different offsets. This can be realized via higher-order modes^34^, or dispersive couplings^35^, yet only partial suppression of the overall periodicity can be achieved or constrained by the inherent trade-off between performance and footprint^36^.

Here, mirroring the effect of the three-body problem^37^, we report a PIC-based spectrometer featuring an disordered photonic molecular system. The concept of photonic-molecule spectrometer was firstly developed by Tsang et al. in 2023, utilizing two cascaded identical microring resonators^35^. In our work, the photonic molecule (PM) employs the interference between the whispering-gallery-modes (WGM) supported by *N* *≥* 3 differing microdisk photonic atoms (PA). Leveraging the reinforced wavelength dependence induced by the mode-hybridization effect in *N*-body-like heteronuclear PMs, a large quantity of disordered and chaotic photonic molecular orbitals are formed generating a response matrix with highly disordered behaviors and a suppressed periodicity lower than 0.11. Finally, combined with a computational reconstruction algorithm^38^, we experimentally demonstrate a miniaturized spectrometer with an ultra-high spectral resolution of 8 pm and a wide operation bandwidth larger than 100 nm. A high bandwidth-to-resolution ratio of 1.25 × 10^4^ and a record low resolution-footprint-product of 28 nm·μm^2^ are obtained in an ultra-compact footprint measuring 70 × 50 μm^2^ with merely a single spatial channel. This device provides a platform for dramatic advancement of in-situ applications requiring ultra-high resolutions in the broadband range.

## Results

### *N*-body photonic molecular system

The three-body problem can be traced back to 1687^39^, in Newton’s descriptions of astronomical systems involving more than two interacting gravitational sources and led Poincaré to the first developments of modern chaos theory^40^. While a two-body system is analytically solvable, the trajectories of the three-body system are typically chaotic, without periodicity and highly sensitive to initial conditions, with a small set of exceptional cases such as the Euler-Lagrange, and BHH family^41^. Inspired by the three-body problem, we build an *N*-body-like PM system to effectively generate a disordered spectrum while maintaining ultra-high Q-factors in a compact footprint, providing a new route to high-performance on-chip spectrometers.

**Fig. 1:**
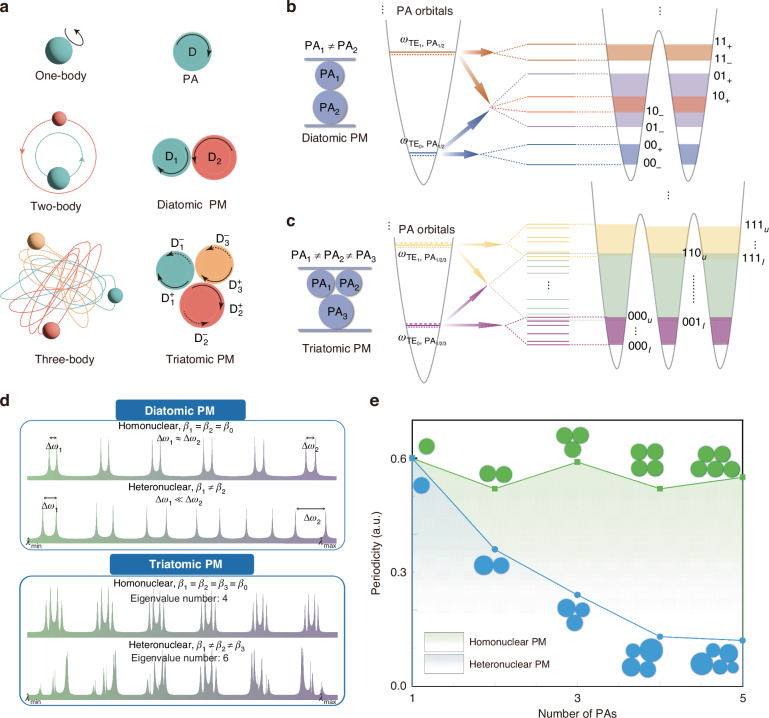
Three-body-like disordered Photonic molecular system. **a** one-body, two-body, and three-body gravitational systems, and the corresponding, analogous photonic atomic or molecular systems. **b** Photonic molecular orbitals formation due to the splitting mechanism of heteronuclear diatomic PMs. From 00_–_ to 11_+_, the first and second numbers represent WGM orders in PA_1_ and PA_2_, respectively, and the subscript +(–) denotes the anti-symmetric and symmetric super-modes, respectively. **c** Photonic molecular orbitals formation due to the splitting mechanism in three-body-like heteronuclear triatomic PMs. From 000 _*l*_ to 111 _*u*_, the first, second and third numbers represent WGM orders in PA_1_, PA_2_ and PA_3_, respectively, and the subscript *u* and *l* denotes molecular orbitals with the lowest and highest energy level. **d** Spectral responses of splitting over wavelength of diatomic homo- and hetero-nuclear PMs, and triatomic homo- and hetero-nuclear PMs. **e** Periodicity as a function of the number of PAs in homo- and hetero-nuclear PMs

Optical micro-resonators are a promising platform for the generation, examination, and application of confined photon states, similar to the confined electron states observed in atoms^42^. The microdisk supports multiple rotationally-symmetric WGMs that are purely confined by the outer medium interface. In this system, an individual microdisk cavity can intuitively be thought of as a PA, where WGM resonances are analogous to the energy levels of atomic orbitals. For a single microdisk PA, the WGMs possess different propagation constants, which correspond to wavelength-dependent spectral features such as their resonance wavelength and FSR, resulting in discrete PA energy levels (See Supplementary Information S1). When PAs are arranged in proximity, the electromagnetic coupling between them gives rise to PM systems. By combining multiple PAs with different radii and coupling gaps (both between atoms and bus waveguides), these systems can provide quasi-chaotic transmission spectra, with distributions of peaks of increasingly variable intensity and random periodicity as more unique PAs are added (see Fig. 1a).

Figure 1b illustrates the formation of photonic molecular orbitals in a diatomic heteronuclear PM with distinct PAs. (see Supplementary Information S1 for diatomic homonuclear PM consisting of two identical PAs for comparison). In the diatomic PM, when interfering with adjacent PAs through evanescent field coupling, individual WGMs split into blue-shifted anti-symmetric super-modes and red-shifted symmetric super-modes (see Supplementary Information S1 for the field profiles of super-modes) due to constructive and destructive interference. This is analogous to the bonding and anti-bonding molecular orbitals in diatomic molecules. Mathematically, the Hamiltonian, **H**, of a diatomic PM supporting a single-mode can be derived from time evolution equations as

1 $${\bf{H}}=\left[\begin{array}{cc}{\beta }_{1} & {\mu }_{12}\\ {\mu }_{12}^{* } & {\beta }_{2}\end{array}\right]$$

Where ${\beta }_{n}={\omega }_{n}-i{\gamma }_{n}$ denotes the original resonant frequency and round-trip optical loss of the PA and *μ*_*mn*_ refers to the inter-cavity coupling strength between the *m*^th^ and *n*^th^ PAs. In the homonuclear PM ${\beta }_{1}={\beta }_{2}={\beta }_{0}={\omega }_{0}-i{\gamma }_{0}$, splitting strengths are linearly proportional to *μ*_12_ as

2 $$\Delta {\omega }_{{\rm{homo}}}=\mathrm{Re}\left({\widetilde{\omega }}_{+}-{\widetilde{\omega }}_{-}\right)=2{\mu }_{12}$$

and remain relatively constant regardless of wavelength change, where ${\widetilde{\omega }}_{\pm }$ denote the solved eigenvalues of the Hamiltonian. Over a large wavelength range, the splitting strength difference is negligible $\left(\Delta {\omega }_{{\rm{homo}},2}\approx \Delta {\omega }_{{\rm{homo}},1}\right)$, as demonstrated in the first row of Fig. 1d. For a heteronuclear diatomic PM, the original resonance wavelengths of WGMs in different PAs exhibit distinct values $({\omega }_{1}\ne {\omega }_{2})$ because of differing propagation constants. Assuming equivalent optical loss coefficients $({\gamma }_{1}={\gamma }_{2})$ in the two PAs, the splitting strength can be estimated as:

3 $$\Delta {\omega }_{{\rm{hetero}}}=2\sqrt{{\left(\frac{{\omega }_{1}-{\omega }_{2}}{2}\right)}^{2}+{\mu }_{12}^{2}}$$

which is determined by the initial resonant wavelengths and inter-cavity coupling, *μ*_12_, simultaneously. As demonstrated in the second row of Fig. 1d, the splitting depth is strengthened significantly $(\Delta {\omega }_{{\rm{hetero}},2}\gg \Delta {\omega }_{{\rm{hetero}},1})$ as ${\omega }_{1}-{\omega }_{2}$ is highly wavelength-dependent. This wavelength dependence in heteronuclear PMs is fundamental to de-periodization (see Supplementary Information S1 for derivation).

We proceed to apply these calculations to triatomic PMs comprising three PAs coupled in a triangular arrangement. The formation of optical molecular orbitals in a heteronuclear triatomic PM is shown in Fig. 1c. The input light generates a dual set of circulating modes (one clockwise, CW, and one counter-clockwise, CCW) within each PA. The distinguishability of CW and CCW modes allows for further releasing of degeneracy and the formation of a greater number of super-modes. The Hamiltonian matrix in a triatomic PM with triangular configuration is expressed as

4 $$\left[\begin{array}{cccccc}{\beta }_{1}^{+} & 0 & 0 & {\mu }_{12} & 0 & {\mu }_{13}\\ 0 & {\beta }_{1}^{-} & {\mu }_{12} & 0 & {\mu }_{13} & 0\\ 0 & {\mu }_{12}^{* } & {\beta }_{2}^{+} & 0 & 0 & {\mu }_{23}\\ {\mu }_{12}^{* } & 0 & 0 & {\beta }_{2}^{-} & {\mu }_{23} & 0\\ 0 & {\mu }_{13}^{* } & 0 & {\mu }_{23}^{* } & {\beta }_{3}^{+} & 0\\ {\mu }_{13}^{* } & 0 & {\mu }_{23}^{* } & 0 & 0 & {\beta }_{3}^{-}\end{array}\right]$$

where the sign +(–) denotes the CW (CCW) circulating modes. In general, six eigenvalues ${\widetilde{\omega }}_{1} \sim {\widetilde{\omega }}_{6}$ can be solved, indicating six splitting resonance peaks in the spectral response. When examining homonuclear conditions only four super-modes are observable in the drop port transmission spectrum rather than six, as demonstrated in the third row of Fig. 1d. This phenomenon occurs due to two of the super-modes behave as nonradiative ‘dark’ states^43,44^. In a heteronuclear triatomic PM, such mode degeneracy is completely removed due to the breaking of spatial symmetry. Consequently, the number of distinguishable, splitting-generated resonance peaks equals that of the calculated eigenvalues. A total of six resonance peaks are observed in the transmission spectrum, as visualized in the last row of Fig. 1d. The wavelength dependence resulting in the splitting strengths and spectral features evolve significantly as the wavelength changes. Compared to heteronuclear diatomic PMs, the triatomic PM generates significantly more complex and disordered spectral features.

It should be noted that, the above calculation only considers inter-cavity interactions of single-modes, yet dozens of WGMs are supported in each PA, inter-cavity WGMs can interfere with the same and different orders, leading to splitting of molecular orbitals by a number that far exceeds the number of PAs present, and too complicated to describe in Hamiltonian matrix. In this process, degeneracy persists in homonuclear PMs (see Supplementary Information S1 for further discussion), but for heteronuclear PMs, dramatic inflation in the number of molecular orbitals occurs, enhancing resonance density, and leading to an improvement in the overall spectral sharpness and diversity. Discussion about the effect of inter-cavity coupling strengths is provided in Supplementary Information S3.

To better illustrate the phenomenon of periodicity suppression/disorder enhancement as the number of distinct PAs increases, a three-dimensional finite-difference time-domain (3D-FDTD) method is utilized to simulate the spectral response of homonuclear and heteronuclear PMs comprising of one to five microdisk PAs. The configuration and topology of PMs are marked in Fig. 1e. Auto-correlation is used as a basis to quantitatively evaluate the periodicity of the PMs. We use the maximum value in the normalized auto-correlation function after falling from the initial value of 1 as the figure-of-merit (FOM) to evaluate the periodicity of the spectra. The periodicity of homo- and heteronuclear PMs with one to five PA components are calculated and plotted in Fig. 1e (see Supplementary Information S5 for transmission spectra and auto-correlation functions). Increasing the number of PAs in homonuclear PMs has almost no suppression on the periodicity, due to the wavelength-dependence that only rely on the coupling strength dispersion is weak and even negligible for our microdisk photonic atoms. However, for the tetratomic or pentatomic heteronuclear PM, integration of distinct PAs results in significant growth in the number of optical super-modes and the variation in geometry and topology breaks spatial symmetry and fully lifts mode degeneracy. In this way, periodicity can be significantly suppressed (see Supplementary Information S7 for more characterization). Further discussion of systematic investigation for PM configurations and coupling gaps can be found in Supplementary Information S6.

### Disordered PM spectrometer

**Fig. 2:**
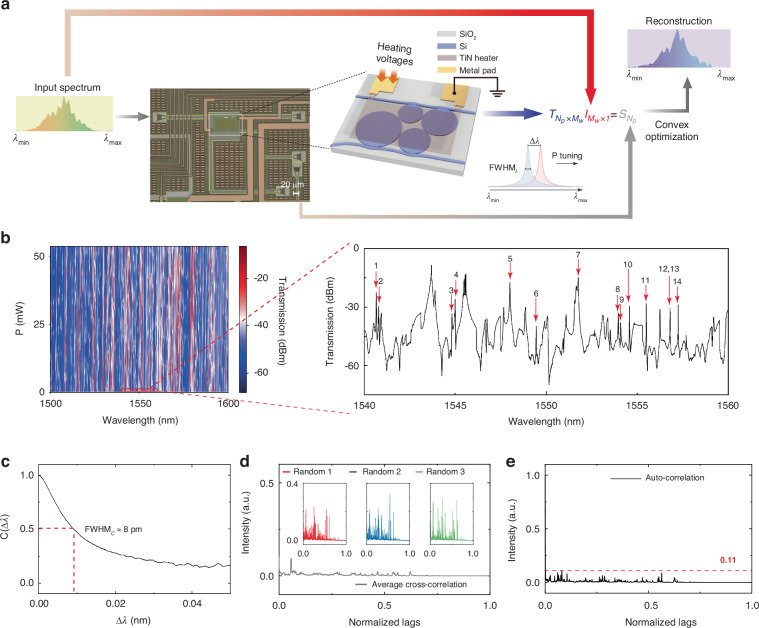
Calibration and characterization of disordered PM spectrometer. **a** The spectral reconstruction process. The inset shows optical microscopy of the fabricated device, while the scale bar indicates a length of 20 μm. **b** Response matrix under linearly swept heating power from 0 to about 53.5 mW. The zoomed section shows transmission spectrum from 1540 nm to 1560 nm in the absence of heating. Red arrows and numbers mark the high-Q resonance peaks detailed in Table. S1. **c** Estimation of the reconstruction resolution, *C*(Δ*λ*), around Δ*λ* = 0. Red dashed line depicts the estimated resolution. **d** Cross-correlation function. The average cross-correlation function is plotted as the black line. Insets show the cross-correlation functions of three groups of transmissions that are randomly picked, labeled as red, blue, and green. **e** Auto-correlation function. Red dashed line shows the periodicity level of 0.11

The layout of the disordered spectrometer based on a heteronuclear tetratomic PM and a schematic of the corresponding reconstruction process is shown in Fig. 2a. The four microdisk atoms have radii of 10 μm, 14 μm, 13 μm, and 18 μm, respectively. The gaps between the microdisks and bus waveguides are randomly set around 180 nm to increase diversity in the coupling strengths. The inset in Fig. 2a shows an optical micrograph of the fabricated device, where the functional microdisk region has a footprint of only 70 × 50 μm^2^. Integration of a TiN heater on top of the microdisks and coupling regions allows for modulation of the PM via thermo-optic (TO) effects (see Supplementary Information S8 for optical microscopy photos with and without heater).

For the computational reconstruction process, a response matrix **T**, with *N*_*p*_ rows, each corresponding to a discrete heating power, and *M*_*w*_ columns, each corresponding to a wavelength band, is pre-calibrated by measuring transmission spectra in the output port while linearly sweeping the external heating power. If the unknown incident signal **I** with *M*_*w*_ wavelength points pass through the PM spectrometer, the spectral information can be sampled and encoded, along with the detected output power **S** with *N*_*p*_ heating channel number recorded. **S** can be mathematically expressed as a vector product:

5 $${{\bf{S}}}_{{N}_{p}\times 1}={{\bf{T}}}_{{N}_{p}\times {M}_{w}}{{\bf{I}}}_{{M}_{w}\times 1}$$

By measuring *N*_*p*_ optical powers, where *N*_*p*_ is far less than the Nyquist frequency $({N}_{p}\ll {M}_{w})$, a signal with *M*_*w*_ wavelength points can be reconstructed. By solving this inverse problem via a reconstruction algorithm (see Methods), we can arrive at an approximation of **I**.

The insets of Fig. 2a shows the fundamental requirement for achieving spectral resolution in two wavelength channels *λ*_2_ and *λ*_1_, known as wavelength-channel decorrelation, is articulated by the requirement that two wavelengths gap Δ*λ* exceeds the full width at half maximum (FWHM) of the power/spectral scanning trace induced by the thermo-optical tuning effect in silicon photonics^35^. As the spectral FWHM is determined by the Q-factor in the resonant structure, higher Q will lead to higher spectral resolution. Figure 2b exhibits the measured response matrix under varying heating power, with the inset showing transmission from 1540 nm to 1560 nm under zero heating bias with a wavelength tuning grid of 1 pm (See Methods for calibration process and refer to Supplementary Information S9 for zoom-in response matrix and more transmission information). Furthermore, the tuning efficiency is analyzed refers to Supplementary Information S10. The calculated Q-factors and bandwidths of specific resonance peaks marked by red arrows and numbers are summarized in Table. S1. As the coupling and interference effects in our tetratomic heteronuclear PM are highly complex, Q-factors for each resonance peak vary significantly. Within the spectral range spanning from 1540 nm to 1560 nm, the loaded Q-factor value for our PM reaches a maximum of 7.74 × 10^5^; such high Q-factors are the foundation for achieving high reconstruction resolution (See Supplementary Information S2 for Q-factor analysis). The estimated resolution based on the response matrices can be numerically calculated by:

6 $$C\left(\Delta \lambda ,N\right)=\frac{\left\langle T\left(\lambda ,N\right)T\left(\lambda +\Delta \lambda ,N\right)\right\rangle }{\left\langle T\left(\lambda ,N\right)\right\rangle \left\langle T\left(\lambda +\Delta \lambda ,N\right)\right\rangle }-1$$

where $T\left(\lambda ,N\right)$ refers to spectral transmission at the *N*^th^ bias for input wavelength *λ*, Δ*λ* is the spectral spacing of two wavelength points, and $\left\langle \cdots \right\rangle$ refers to the average over *λ*. $C(\Delta \lambda ,N)$ is plotted in Fig. 2c. The estimated reconstruction resolution is full width at half maximum (FWHM) of $C(\Delta \lambda ,N)$ which is approximately 8 pm. The calculated resolution largely conforms to the average value of Q-factors of numerous resonance peaks across the whole spectrum.

We further use cross-correlation to identify the degree of independence between any two transmission spectra of the measured response matrix. The calculated average cross-correlation is marked as black line, and the other three cross-correlations of arbitrarily picked three pairs of transmission spectra are marked as red, blue, and green scatter dots respectively in Fig. 2d. It is observed that the average cross-correlation is maintained at a low level below 0.1, indicating a nearly mutually orthogonal response matrix. Besides, different WGMs have different mode distributions, which lead to different effective refractive index, rendering different thermo-optical sensitivity. Inter-cavity coupling *μ*_*mn*_ is strongly manipulated between different channels, resulting in spectral signatures that vary with power channels, which is far beyond merely resonant red shifting (see Supplementary Information S11). The implementation of this mode evolution yields an impressive benefit in terms of the orthogonality of the response matrix. The average auto-correlation is also computed and presented in Fig. 2e. The periodicity, as expressed by the autocorrelation figure of merit, is below 0.11, which is indicated by a red dashed line, demonstrating a nearly fully-suppressed periodicity.

### Experimental results

**Fig. 3:**
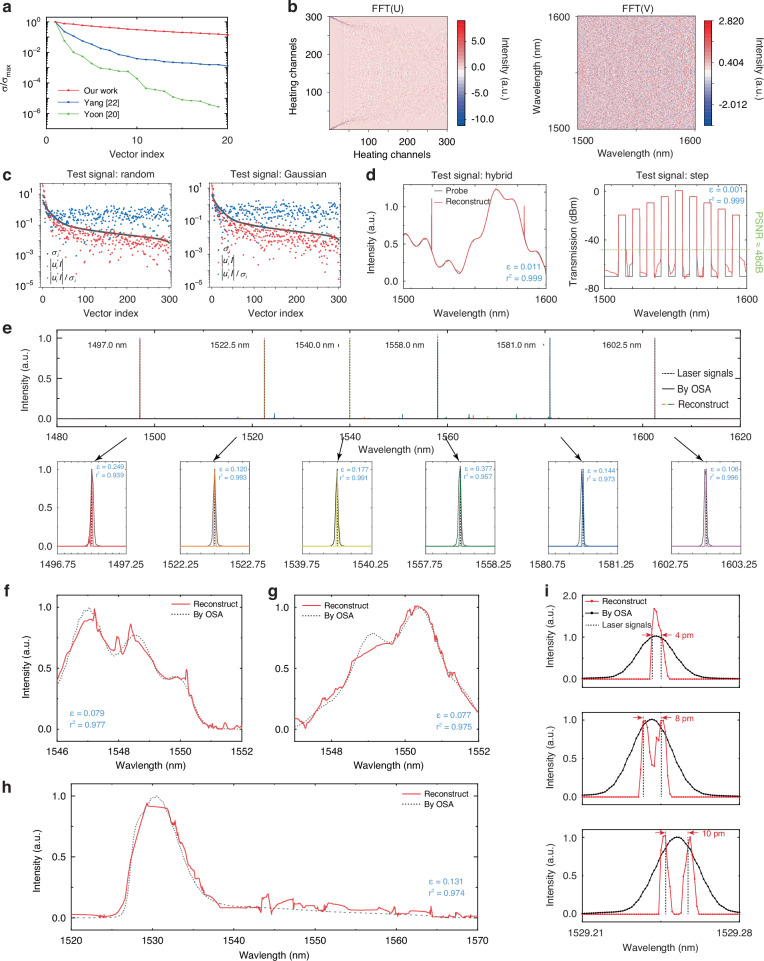
Theoretical analysis and reconstruction results. **a** Calculated singular value (${\sigma }_{{\rm{i}}}/{\sigma }_{\max }$) of experimentally pre-calibrated response matrix **T** of PM spectrometer (red lines) and other published works (blue and green lines), respectively. **b** Calculated Fast Fourier Transform (FFT) of left singular vector (**U**) and right singular vector (**V**) of ***T***. **c** *Picard plots* including singular value ${\sigma }_{{\rm{i}}}$_,_ SVD coefficients ${u}_{i}^{* }{\bf{I}}$ and solution coefficients ${u}_{i}^{* }{\bf{I}}/{\sigma }_{i}$ of random and Gaussian signals. **d** Numerical test of hybrid signal and step signal. The relative errors (*ε*) and coefficients of determination (*r*^2^) are labeled. Demonstrations of the spectrometer’s reconstruction capabilities with respect to **e** narrow, monochromatic peaks over the full operational range of the device; **f**, **g** continuous signals with diverse features; **h** broadband continuous signal; **i** the minimum distinguishable peak separation of 8 pm

We now numerically analyze the experimentally pre-calibrated response matrix **T**, and the effectiveness of the proposed spectrometer in retrieving various probe signals with distinct optical features is numerically assessed. When processing in real-world application with measurement noise, prior knowledge or assumptions are demanded as regularization terms. For evaluating the quality of our response matrix, which reflects how much the aid of prior knowledge in the decoding is needed, singular-value decomposition (SVD) is exploited on matrix **T**. By utilizing SVD, pseudo-inverse of **I**, marked as **I**^†^ can be expressed as^45^

7 $${{\bf{I}}}^{\dagger }=\mathop{\sum }\limits_{i=1}^{{N}_{p}}\frac{{u}_{i}^{* }{\bf{S}}}{{\sigma }_{i}}{v}_{i}$$

where the diagonal entries *σ*_*i*_ denotes the singular value of **T**, formed in descending order; *u*_*i*_ and *v*_*i*_ are *i*th left- and right- singular vector. We use the decay rate of the singular values to describe the quality of the response matrix. ${{\sigma }_{i}}/{{\sigma }_{\max }}$ as a function of vector index *i* is illustrated in Fig. 3a, marked as red line. Compared to other works marked as blue and green lines, the descending rate of our work exhibits an extradentary decrease, indicating a robust response matrix against noise. The orthogonality of the response matrix is assessed by the condition number $\kappa \left({\bf{T}}\right)={{||}{\bf{T}}{||}}_{2}{{||}{{\bf{T}}}^{{{\mbox{-}}}{\mathbf{1}}}{||}}_{2}={\sigma}_{\max }/{\sigma}_{\min }$. If considering measurement noise *δS*, the degree of ${{\bf{I}}}^{\dagger }$ are affected by noise is demonstrated as:

8 $$\frac{{||}{{\bf{I}}}^{\dagger }-{\bf{I}}{||}}{{||}{\bf{I}}{||}}\le \kappa \left({\bf{T}}\right)\frac{{||}\delta {\bf{S}}{||}}{{||}{\bf{S}}{||}}$$

Consequently, a large condition number indicates that slight perturbation in the detected optical power **S** can have a substantial impact on the reconstruction results. Our condition number of the PM spectrometer is 5.416 × 10^2^, which is two to three orders of magnitude smaller than another two published works based on the reconstruction spectrometer as summarized as follows.

**Table:** 

Resource	*κ*(T)
Yang^22^	1.474 × 10^4^
Yoon^20^	3.631 × 10^5^
Our work	5.416 × 10^2^

Both the decay rate of singular values and condition number of our PM spectrometer indicate a quasi-orthogonality of each transmission spectra under heating power channels, demonstrating exceptional robustness against measurement noise and high accuracy and stable reconstruction capabilities. Furthermore, Fast Fourier Transform (FFT) of the left singular vector (FFT(**U**)) and right singular vector (FFT(**V**)) are shown in Fig. 3b. FFT(**U**) has a wide distribution of components from low to high frequency, making it possible to collect all information with fewer channels. FFT(**V**) exhibits random intensity distributions, suggesting the highly randomization of response matrix **T**^36^. To validate the existence of a solution for the linear inverse problem, we calculate *Picard plots* with the behavior of SVD coefficients $$\begin{array}{c}{u}_{i}^{* }{\bf{I}}\end{array}$$, solution coefficients ${u}_{i}^{* }{\bf{I}}/{\sigma }_{i}$, along with singular values for Gaussian and random signals as shown in Fig. 3c. Red dashed line suggests the decay rate of SVD coefficients. Clearly, the discrete Picard condition is satisfied, as evidenced by the faster decay of SVD coefficient than the singular values initially until they level off for $i\ge 50$ at a plateau determined by noise, or by the initial decay of the solution coefficients and the absence of apparent increase even at a high index. This indicates a bounded solution with practical norm and a convergent solution can be realized^46^. (See the test signals and more *Picard plots* in Supplementary Information S12). Numerical calculations are performed to assess the efficacy of the proposed spectrometer in retrieving various signals including a hybrid signal consists of smooth and discrete signals and a step signal. The probe signals and reconstructed signals are plotted in Fig. 3d, marked as black and red lines, respectively. We mathematically generate probe signals of *M*_*w*_ wavelength points then feed into the measured response matrix **T**. The reconstruction accuracy is evaluated with relative error ($\varepsilon =\frac{{{||}{{\bf{I}}}^{\dagger }-{\bf{I}}{||}}_{2}}{{{||}{\bf{I}}{||}}_{2}}$) and coefficient of determination (${r}^{2}=1-\frac{{\sum }_{i=1}^{n}{({{\rm{I}}}_{i}^{{{\dagger }}}-{{\rm{I}}}_{i})}^{2}}{{\sum }_{i=1}^{n}{({{\rm{I}}}_{i}-{\bar{{\rm{I}}}}_{i})}^{2}}$) and are labeled in Fig. 3d (See more numerical results and reconstruction algorithm in Supplementary Information S13).

We now experimentally demonstrate the performance of the disordered PM spectrometer with respect to a range of different characterization challenges. For single peak reconstruction, we use a tunable laser source as input signals at different wavelengths from 1497 nm to 1603 nm, throughout the whole operation bandwidth of more than 100 nm. The reconstruction results for these single narrow linewidth signals are presented in Fig. 3e. Signals located over the whole bandwidth are successfully reconstructed throughout the whole operation bandwidth, with a signal-to-noise ratio (SNR) of approximately 15 dB (for SNR exploration within a narrower operational bandwidth, see Supplementary Information S14). Zoom-in spectra are also presented in Fig. 3e. Bandwidth of these laser signals is ultra-narrow of 60 kHz, hence can be regarded as discrete signals, labeled as dashed black lines. The calculated average relative errors (*ε*) and coefficient of determination (*r*^2^) of these resolved spectra is 0.193 and 0.975, respectively, indicating that signals located over the whole bandwidth are successfully reconstructed with high positional accuracy with an overall FWHM of 10 pm. Furthermore, a commercial optical spectrum analyzer (OSA, Yokogawa AQ6370C) is utilized to measure these single-peak signals for reference, which is presented as dashed lines in Fig. 3e. Given that the highest resolution achievable by the OSA is 20 pm, the FWHMs of single peaks measured by OSA is about 20 pm, about twice that of our PM spectrometer. It’s noted that the bandwidth in this PM system is physically limited by the material systems, and experimentally limited by the spectral response of the grating couplers (GC) and the measuring instruments. For continuous signal reconstruction, we use the OSA to measure the optical response of the signal that is coded by wave-shaper in advance for calibration and reference, as black dashed line in Fig. 3f–h while the reconstructed results by the PM spectrometer are exhibited in red line. For the continuous signals with diverse spectra features as exhibited in Fig. 3f, g, the calculated *r*^2^ are 0.977, 0.975, and *ε* are 0.079, 0.077, respectively. For the broadband continuous signals as exhibited in Fig. 3h, the calculated *r*^2^ is 0.974 and *ε* is 0.131. (See experimental details in Methods). More reconstruction results of other representative continuous signals such as Gaussian signals and bandpass signal are provided in Supplementary Information S15. The primary cause of disagreement with reference in continuous signal reconstruction stems from the inevitable error in the discretization process and the inherent limitation of compressive sensing that continuous signals violate sparsity principle. Because of widely distributed high-Q resonant peaks in our PM spectrometer, the real transmission varies significantly around the resonant wavelength, which cannot be recorded when sampling with coarse wavelength gird. This dilemma can be alleviated by increasing sampling wavelength points with a smaller grid, however, increases the demand for sampling channels and calculation burden significantly in turn (See details in Supplementary Information S16 and S13).

We further explore the resolution limits of our device by simultaneously launching two laser signals at different wavelengths. Figure 3i demonstrates the reconstruction result when two laser signals are separated by 4 pm, 8 pm, and 10 pm with black dashed lines demonstrating the laser signals. Reference spectra measured by OSA is plotted in black solid lines. It can be seen that 4 pm is beyond the resolution limit, and the PM spectrometer recognizes it as a single peak. When the two signals’ wavelength distance increases to 8 pm, two peaks can be evidently distinguished, demonstrating an ultra-high resolution of 8 pm that satisfies the Rayleigh criterion. When the two signals’ wavelength distance further increases to 10 pm, the discrete signals can be fully reconstructed as separate peaks. Because these two closely spaced peaks, possessing a wavelength separation of less than 20 pm, is beyond the resolution limits of the OSA, the OSA recognizes these dual-peak signals as single peak characterized by a smooth Lorentzian shape. Generally, optical noises such as spatial oscillation of multi-axis manual stages and fibers, and electrical noises contribute to the overall reconstruction errors. Optical noises can be effectively suppressed by the utilization of optical packaging. The utilization of temperature controllers is also considered in our future measurement for mitigating reconstruction errors caused by temperature fluctuations. (see Supplementary Information S17 for reconstruction provided by alternatively fabricated device). Additionally, we further investigate more characteristics of spectral reconstruction. Stability is discussed and detailed in Supplementary Information S18. Transmissions after 5 operations, 10 operations, and 14 days mirror initial transmission, signifying good mechanical stability. Single peak signals are successfully resolved under these conditions, although a few picometers wavelength shift generated due to the ambient temperature differences, which can be compensated by utilizing the temperature controller. The exploration of sensitivity is conducted indicating a resolvable power level lower than –30 dBm, which is provided in Supplementary Information S19.

## Discussion

The performance of computational spectrometers is primarily governed by the properties of the spectral response matrix encoded in their detection system, in particular, the sharpness and orthogonality of their features. By exploiting the chaotic interactions in disordered photonic molecule systems, we have designed and demonstrated a device that can be produced straightforwardly through standard PIC fabrication processes and which can controllably generate pseudo-random response matrices with dramatically nullified periodicity and ultra-high Q-factors. Through this, we showcase a platform for highly efficient computational spectroscopy in an ultra-miniaturized, single-channel form factor of 70 × 50 μm^2^; the device shows a high bandwidth-to-resolution ratio (BRR) of 1.25 × 10^4^ and a record low resolution-footprint-product (RFP) of 28 nm·μm^2^.

**Fig. 4:**
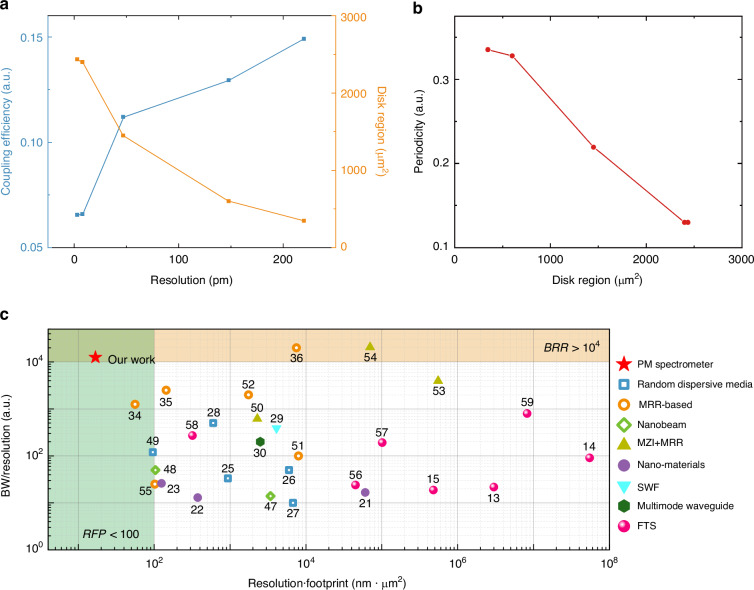
Analysis and performance comparison. **a** Average coupling efficiencies and microdisk covered regions of other alternative devices as functions of estimated resolution. **b** Periodicity of other alternative devices as functions of microdisk-covered regions. **c** Performance comparison. MRR is the abbreviation for microring-resonator; MZI is the abbreviation for Mach-Zehnder Interferometer; SWF is the abbreviation for stratified waveguide grating

To conduct a more comprehensive investigation into the deterministic impact of PM structure parameters on spectral resolution, we additionally fabricated and analyzed alternative spectrometer devices based on heteronuclear PMs with varying coupling efficiencies between microdisks (and bus waveguides) and microdisk sizes. The reduction of average coupling strengths results in a decrease in the external losses of the resonator. Meanwhile, the expansion of microdisk regions leads to an effective increase in optical path length and the number of supported atomic orbitals. As illustrated in Fig. 4a, both the reduction of average coupling strengths and the increase of microdisk regions contribute to an enhancement in the Q-factors, resulting in an optimization of the estimated resolution to 3 pm. Moreover, it can be observed from Fig. 4b that the periodicity in PMs diminishes gradually as microdisk regions increase. This can be attributed to further quantities of super-mode generation based on the increased number of atomic orbitals in microdisks with larger radius.

Finally, we conduct a comprehensive comparison of the primary performances of various state-of-art computational spectrometers^21–23,25–30,34–36,47–55^ and Fourier-transform spectrometers^13–15,56–59^ that have been previously reported in Fig. 4c. Overall, Fourier-transform spectrometers encounter a more prominent three-way trade-off among resolution, bandwidth and footprint than computational spectrometers, especially compared to those based on resonators. We emphasize the superior performance of our spectrometer in terms of resolution, operation bandwidth, and footprint. Because resolution is tightly related to footprint, the evaluation of spectrometer performance per unit footprint is conducted by defining RFP. The evaluation of the trade-off between resolution and bandwidth in a single spectrometer device is performed using BRR, which is one of the most representative spectrometer performance indicators. This ratio is inherently constrained by the contradiction between the larger OPL required for high resolution and the resulting decrease in bandwidth, which are mostly limited to the order of tens or hundreds. As illustrated in Fig. 4c, the proposed PM spectrometer achieves a high value of BRR within the lowest RFP value (See Supplementary Information S21 for more comparison details). Furthermore, compared to spectral-to-spatial mapping spectrometers that unique speckles are generated via multimode interference and acquired from numerous physical channels, sampling channels of PM spectrometer are created in the time domain and only one spatial channel is required, which further reduces physical size and substantially increases the level of on-chip integration. The sampling channels can be flexibly adapted, accommodating various applicable scenarios. Considering the operation bandwidth of 100 nm is mainly limited by GC and measurement instruments, wider operating bands with higher bandwidth-to-resolution ratio can be realized further until material bandgap limit of Silicon-on-Insulator (see Supplementary Information S20 for the design operated in other wavelength bands). This disordered photonic molecule spectrometer design thoroughly bypasses typical three-way trade-off between resolution, operational bandwidth and device footprint seen in miniaturized spectrometer configuration and shows promise for in-situ applications.

## Materials and methods

### Chip fabrication

The tetratomic PM spectrometer is fabricated in Chongqing United Microelectronics Center (CUMEC) using their CSiP180A technology with 248 nm lithography. The multi-project wafer (MPW) starts with silicon-on-insulator (SOI) 200 mm wafers using 220 nm top silicon and 2 μm buried oxide (BOX). After 1 μm oxide cladding 120 nm TiN metal heater and aluminum metal interconnection with a thickness of 1 μm is formed, subsequently.

### Calibration and reconstruction experiments

A pre-calibration process is demonstrated as follows. A tunable continuous wave laser (Santec TSL 770) and a power monitor (Santec MPM 210) are utilized for sampling and data collecting. Broadband grating couplers (GC) with less than 5 dB insertion loss are applied for fiber-chip coupling. A source meter (Keithley 2400) is used to offer an external driving power source. A maximum external power *P*_max_ of 53.5 mW is applied to the heater. For eliminating current fluctuations due to unstable contact, we utilize wire bonding and an electrical package for our fabricated chip. The current value can be stabilized at 1 × 10^-4^ mA after electrical packaging. The dimensions of response function **T** are determined by the wavelength point number ${M}_{w}={BW}/{\rm{\delta }}\lambda$, and ${N}_{p}={P}_{\max }/{\rm{\delta }}P$ mutually, where ${\rm{\delta }}P$ is the sampling step of about 0.178 mW; *BW* is the spectrometer measurable bandwidth (1500 nm to 1600 nm); ${\rm{\delta }}\lambda$ is the wavelength grid of 10 pm. Here, a spectrum with *M*_*w*_ of 10000 points can be reconstructed with merely *N*_*p*_ of 300 heating power channels, demonstrating an ultra-high compress ratio. The pre-calibration process takes approximately 750 s.

For the single peak reconstruction, we use a CW laser source (Santec TSL 770) to generate discrete narrow linewidth signals with wavelengths of 1497 nm, 1522.5 nm, 1540 nm, 1558 nm, 1581 nm and 1602.5 nm and inject them into the input port of PM spectrometer, demonstrating an operation bandwidth larger than 100 nm. For two closely separated discrete signals reconstruction, two narrow linewidth signals with wavelength separation of 4 pm, 8 pm, and 10 pm are produced by two CW lasers which combined with a 3 dB coupler subsequently. For the purpose of exploring resolution, we conduct sampling with wavelength grids of 1 pm here. We sample every 0.5 s with an integration time of 0.1 s. Since the rise time and the fall time are less than 10 μs, sampling starts at 0.3 s after sending commands to the source meter to regulate the external biases, in order to ensure a stable temperature of silicon waveguide.

For continuous signal reconstruction with diverse spectra features, a broadband amplified spontaneous emission (ASE) source is utilized to supply incident continuous light with a bandwidth of over 60 nm centered in 1550 nm. A programmable optical filter (Finisar Wave-shaper 1000 s) is connected to generate the required test signals. An Erbium-Doped Fiber Amplifier (EDFA) is connected after the wave-shaper to amplified signal light. A polarization controller is connected subsequently to adjust the continuous signal to TE mode, and a 3 dB coupler divides the signal into two paths: one is injected into the PM spectrometer while a commercial optical spectrum analyzer (OSA, Yokogawa AQ6370C) recorded spectra from the other path. For the broadband continuous signal reconstruction, an EDFA is connected after a broadband ASE laser source to provide broadband continuous signal of about 50 nm. The spectrum reconstructions are performed by running the CVX optimization algorithm on Matlab, based on a AMD Ryzen 7 3700X CPU with 64 GB memory. ANSYS Lumerical FDTD is used to perform the optical transmission simulations for proposed PM spectrometer.

### Reconstruction algorithms

In computational spectrometers, advanced algorithmic or machine learning techniques are used to reconstruct spectra by to decoding the interaction of the target spectra with the response matrix generated by the spectral encoder system. The response matrix predominantly determines performances of the computational spectrometer in terms of resolution, bandwidth, and reconstruction fidelity. For the reconstruction, there are some general criteria for the response matrix that need to be satisfied simultaneously: (i) Each transmission should be independent of each other, and the response matrix should be sufficiently sharp with strong orthogonality, so that the spectrometer can detect minor spectral features of the input signals with high resolution. (ii) Each transmission should exhibit disorder and randomness with diverse spectral features; as such periodicity should be as low as possible across the operational bandwidth. Non-negligible periodicity, leads to multicollinearity, with redundant information encoded in the response matrix giving rise to large condition numbers; in an ill-conditioned system, slight perturbation in the detected optical power can have a substantial impact on the reconstruction results and a more computationally challenging matrix inversion.

The reconstructed signal ${{\bf{I}}}^{{{\dagger }}}$ can be generated by solving the under-determined linear least-squares:

9 $${{\bf{I}}}^{{{\dagger }}}={{\rm{argmin}}}_{I\in {{\mathbb{R}}}^{+}}{{\rm{||}}{\bf{TI}}-{\bf{S}}{\rm{||}}}_{2}^{2}$$

where ${{||}\cdot {||}}_{2}$ represents the *l*_2_-norm. It is noteworthy to mention that the response matrix obtained in real-world circumstances will surely encounters measurement noise plus a certain level of ill-conditioning because of less sampling points. In order to overcome this overfitting obstacle, the regularization coefficient is introduced as:

10 $${{\bf{I}}}^{{{\dagger }}}={{\rm{argmin}}}_{{\boldsymbol{I}}\in {{\mathbb{R}}}^{+}}\left({{\rm{||}}{\bf{TI}}-{\bf{S}}{\rm{||}}}_{2}^{2}+{\alpha }_{1}{{\rm{||}}{\bf{I}}{\rm{||}}}_{1}+{\alpha }_{2}{{\rm{||}}{\bf{I}}{\rm{||}}}_{2}+{\alpha }_{3}{{\rm{||}}D{\bf{I}}{\rm{||}}}_{2}\right),{\rm{subject}}\, {\rm{to}}\,0\le {{\bf{I}}}_{i}\le 1$$

where ${\alpha }_{1}$ is the weight regularization coefficient for the *l*_1_-norm of input matrix **I**, which is vital in the regression of ${{\bf{I}}}^{{{\dagger }}}$ into discrete untrivial solutions; ${\alpha }_{2}$ is the weight regularization coefficient for the *l*_2_-norm of **I**, which strengthens the robustness against measurement noise and decreases complexity; *D* refers to the first derivative operator; ${\alpha }_{3}$ is the weight regularization coefficient for the *l*_2_-norm of the first derivation of **I**, which is critical for optimizing continuity and smoothness of continuous broad-band signals. Solving this optimization problem with discrete signals typically take 70 s for CVX in Matlab. Optimization for continuous signals basically requires longer iterative time that ups to 6 min.

## Supplementary information

Supplemental material

## Data Availability

All the data and methods needed to evaluate the conclusions of this work are presented in the main text and Supplementary Information. Additional data can be requested from the corresponding author.
