# Supplementary material for: Miniaturized disordered photonic molecule spectrometer
Source: Light Sci Appl. 2025 Mar 31;14:144. doi: 10.1038/s41377-024-01705-w (PMC11958646; doi:10.1038/s41377-024-01705-w)
Supplement: Supplementary file 1 — Supplemental material [file 41377_2024_1705_MOESM1_ESM.docx]

Supplementary Information for

**Miniaturized Disordered Photonic Molecule Spectrometer**

Yujia Zhang^1^†, Tom Albrow-Owen^2^†, Zhenyu Zhao^1^†, Yinpeng Chen^3^†, Yaotian Zhao^1^, Hannah Joyce^2^, Tawfique Hasan^2^*, Zongyin Yang^3^*, Yikai Su^1^*, Xuhan Guo^1^*

^1^State Key Laboratory of Photonics and Communications, School of Information and Electronic Engineering, Shanghai Jiao Tong University, Shanghai 200240, China

^2^Department of Engineering, University of Cambridge; Cambridge, CB3 0FA, UK.

^3^College of Information Science and Electronic Engineering, Zhejiang University; Hangzhou, 310027, China.

*Corresponding author. Email: guoxuhan@sjtu.edu.cn; [yikaisu@sjtu.edu.cn](mailto:yikaisu@sjtu.edu.cn); th270@cam.ac.uk; yangzongyin@zju.edu.cn.

†These authors contributed equally to this work.

**S1. Theoretical analysis of One- and Two-body PMs**

Fig. **S1a** shows microdisk PA with a radius of 10 μm supporting multiple WGMs, corresponding to different AOs. The transverse-electrical (TE) polarization of WGMs from TE_0_ to TE_2_, and TE_10_ are also provided. These WGMs possess different propagation constants, hence spectral features such as resonance wavelength ($\lambda_{\mathrm{res}}=n_{\mathrm{eff}}L_{\mathrm{rt}}/m,m=1,2,3,\ldots$), full width at half maximum (FWHM, $\mathrm{FWHM}=\lambda_{\mathrm{res}}/Q$) and free-spectral range(FSR, $\mathrm{FSR}=\lambda_{\mathrm{res}}^{2}/n_{g}L_{\mathrm{rt}}$), are wavelength-dependent, contributing to the discriminability of atomic energy levels in PA.

The time evolution equations for diatomic PMs that support single-mode are expressed as:

$$\begin{aligned} \begin{aligned} &\frac{d}{dt}D_{1}=i\omega_{1}D_{1}-\gamma_{1}D_{1}-i\mu_{12}D_{2}+\sqrt{k_{1}}S_{\text{in}} \\ &\frac{d}{dt}D_{2}=i\omega_{2}D_{2}-\gamma_{2}D_{2}-i\mu_{12}^{*}D_{1} \end{aligned}\#\left( S1 \right) \end{aligned}$$

where denotes the field amplitude in the *n*th PA, $\omega_{n}$ and $\gamma_{n}$ denote the frequency and total optical loss of the *n*th PA. The coupling strengths between the *m*th and *n*th PAs and between the bus waveguides are defined as $\mu_{mn}$ and *k_n_*, respectively; if the coupling is lossless, $\mu_{mn}=\mu_{mn}^{*}$ is obtained. *S*_in_ refers to the input power. Rewriting equation (S1) into a matrix form $i\frac{d}{dt}\mathbf{D}=\mathbf{MD}-\mathbf{B}$, where $\mathbf{D}=\left[ \begin{aligned} D_{1} \\ D_{2} \end{aligned} \right]$and $\mathbf{B}=\left[ \begin{aligned} -i\sqrt{k_{1}}S_{\mathrm{in}} \\ 0 \end{aligned} \right]$. The coefficient matrix **M**then becomes

$$\begin{aligned} \mathbf{M}=\left[ \begin{matrix} \beta_{1} & \mu_{12} \\ \mu_{12}^{*} & \beta_{2} \end{matrix} \right]-\omega\mathbf{I}=\mathbf{H}-\omega\mathbf{I}\boldsymbol{\#}\left( S2 \right) \end{aligned}$$

where $\beta_{n}=\omega_{n}-\gamma_{n}$ and **H** is the Hamiltonian matrix for a diatomic PM. The solved eigenvalues of the Hamiltonian in general are calculated as

$$\begin{aligned} \tilde{\omega}_{\pm}=\frac{\beta_{1}+\beta_{2}}{2}\pm\sqrt{\left( \frac{\beta_{1}-\beta_{2}}{2} \right)^{2}+\mu_{12}^{2}}\#\left( S3 \right) \end{aligned}$$

where $\pm$ refers to the symmetry and anti-symmetry modes. Based on the eigenvalues, splitting strengths of hetero- and homo-nuclear PM can be calculated, as demonstrated in the manuscript.

In our PMs consisting of microdisk PAs, multiple resonant WGMs are involved in inter-cavity interfering with the same order or different orders in each PA to split into MOs with a number greater than the number of AOs. For easier clarification, we take the diatomic heteronuclear PM as an example. Equation (S1) can be rewritten as eighteen equations if only considering WGMs of the first three orders from TE_0_ to TE_2_ in each cavity:

$$\begin{aligned} \begin{aligned} \frac{d}{dt}D_{\mathrm{TE}_{0},\mathrm{PA}_{1}}{=\omega}_{\mathrm{TE}_{0},\mathrm{PA}_{1}}D_{\mathrm{TE}_{0},\mathrm{PA}_{1}}-\gamma_{\mathrm{TE}_{0},\mathrm{PA}_{1}}D_{\mathrm{TE}_{0},\mathrm{PA}_{1}}-i\mu_{\mathrm{TE}_{0},\mathrm{PA}_{1}&\mathrm{TE}_{0},\mathrm{PA}_{2}}D_{\mathrm{TE}_{0},\mathrm{PA}_{2}}+\sqrt{k_{\mathrm{TE}_{0},\mathrm{PA}_{1}}}S_{\mathrm{in}} \\ \ldots\\ \frac{d}{dt}D_{\mathrm{TE}_{2},\mathrm{PA}_{1}}=i\omega_{\mathrm{TE}_{2},\mathrm{PA}_{1}}D_{\mathrm{TE}_{2},\mathrm{PA}_{1}}-\gamma_{\mathrm{TE}_{2},\mathrm{PA}_{1}}D_{\mathrm{TE}_{2},\mathrm{PA}_{1}}-i\mu_{\mathrm{TE}_{2},\mathrm{PA}_{1}&\mathrm{TE}_{2},\mathrm{PA}_{2}}D_{\mathrm{TE}_{0},\mathrm{PA}_{2}} \\ \frac{d}{dt}D_{\mathrm{TE}_{0},\mathrm{PA}_{2}}=i\omega_{\mathrm{TE}_{0},\mathrm{PA}_{2}}D_{\mathrm{TE}_{0},\mathrm{PA}_{2}}-\gamma_{\mathrm{TE}_{0},\mathrm{PA}_{2}}D_{\mathrm{TE}_{0},\mathrm{PA}_{2}}-i\mu_{\mathrm{TE}_{0},\mathrm{PA}_{1}\text{\& TE}_{0},\mathrm{PA}_{2}}^{*}D_{\mathrm{TE}_{0},\mathrm{PA}_{1}} \\ \ldots\\ \frac{d}{dt}D_{\mathrm{TE}_{2},\mathrm{PA}_{2}}=i\omega_{\mathrm{TE}_{2},\mathrm{PA}_{2}}D_{\mathrm{TE}_{2},\mathrm{PA}_{2}}-\gamma_{\mathrm{TE}_{2},\mathrm{PA}_{2}}D_{\mathrm{TE}_{2},\mathrm{PA}_{2}}-i\mu_{\mathrm{TE}_{2},\mathrm{PA}_{1}\text{\& TE}_{2},\mathrm{PA}_{2}}^{*}D_{\mathrm{TE}_{2},\mathrm{PA}_{1}} \end{aligned}\#\left( S4 \right) \end{aligned}$$

In homonuclear PMs, $\omega_{\mathrm{TE}_{n},\mathrm{PA}_{1}}=\omega_{\mathrm{TE}_{n},\mathrm{PA}_{2}}$, and $\mu_{\mathrm{TE}_{m},\mathrm{PA}_{1}\&\mathrm{TE}_{n},\mathrm{PA}_{2}}=\mu_{\mathrm{TE}_{n},\mathrm{PA}_{1}\&\mathrm{TE}_{m},\mathrm{PA}_{2}}$ are established where *m* ≠ *n*. Therefore, the significant reduction in the number of equations decreases the number of eigenvalues, which indicates a considerable decrease in the generation of super-modes in the spectrum. However, this degeneracy is fully lifted in heteronuclear PMs for the breaking of the two equalities mentioned above. Fig. **S1b** illustrates the optical MO formation of homonuclear PM with two identical PAs, where the +(–) represents the anti-symmetry and symmetry modes, respectively. Because of the same energy level of $\omega_{\mathrm{TE}_{n},\mathrm{PA}_{1}}$ and $\omega_{\mathrm{TE}_{n},\mathrm{PA}_{2}}$, super-modes *mn*$\pm$ and *nm*$\pm$ (the first and second number denotes the WGM orders in PA_1_ and PA_2_ respectively) are degenerate and indistinguishable from the spectrum.

The generated magnetic field profile of super-modes of 00$\pm$, 01$\pm$ and 11$\pm$ in heteronuclear diatomic PM are depicted in Fig. **S1c.**


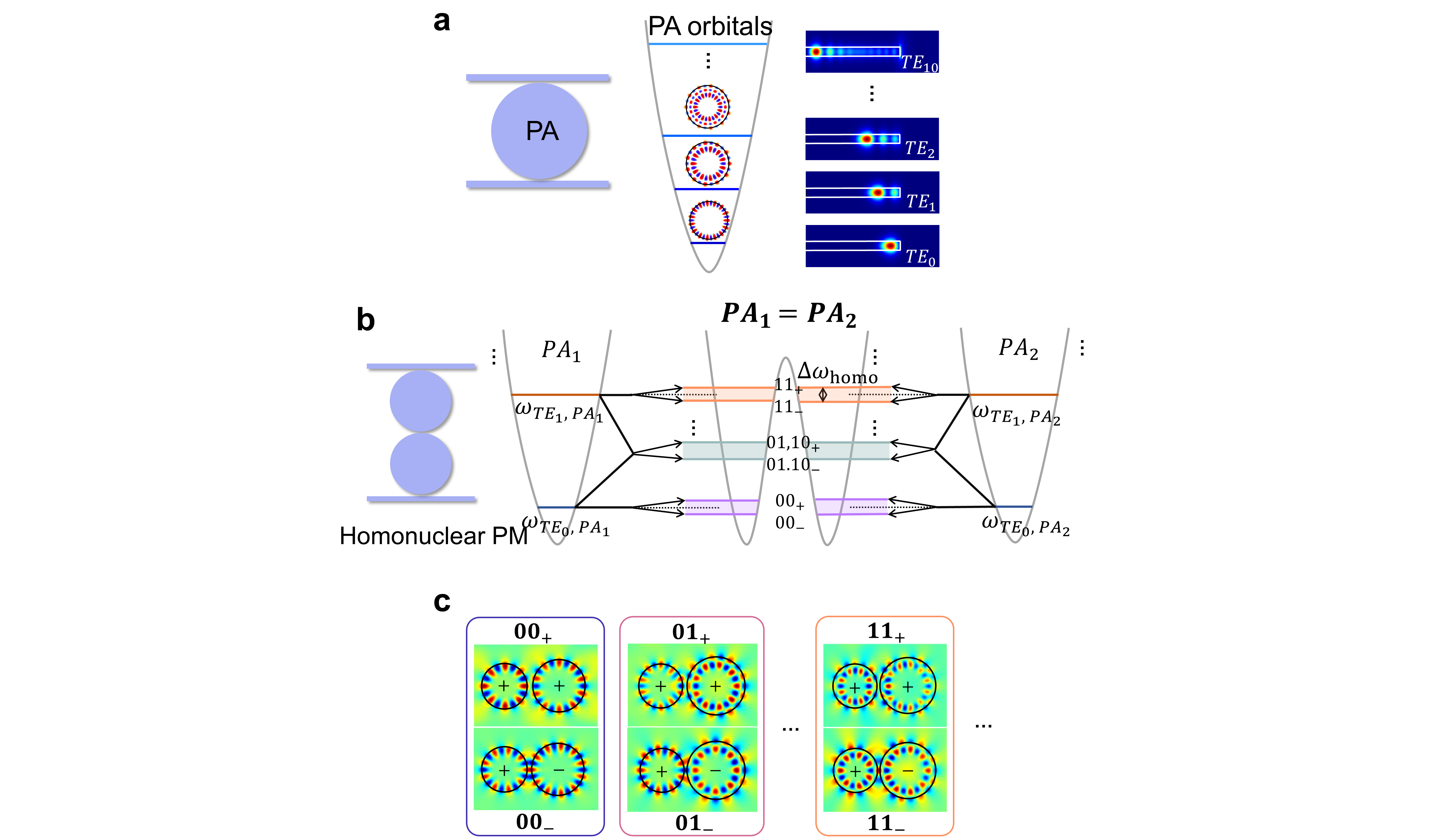


**Fig. S1 | PM system with one and two PAs. a**, Microdisk PA supporting WGMs of TE_0_, TE_1_, TE_2_, and TE_10_, corresponding to photonic AOs with different energy levels. **b**, Photonic MOs formation due to splitting mechanism of homonuclear diatomic PMs. **c,** Magnetic field profiles of super-modes of 00$\pm$, 01$\pm$ and 11$\pm$.

**S2. Analysis of the Q-factors**

As marked by red arrows and numbers in Fig. **2b** of the manuscript, the calculated Q-factors and bandwidths of specific resonance peaks are summarized in Table. **S1**. There are multiple reasons for these high Q-factors. Firstly, the microdisk facilitates the propagation of WGMs that are entirely confined by the surface of the outer medium because of the total internal reflection of light. Microdisk cavities consistently demonstrate higher Q-factors in comparison to microring resonators of similar OPLs. This is primarily attributed to the significant decrease in Rayleigh scattering loss, which arises from the existence of only one rough sidewall during fabrication. Secondly, as proved by Boriskina^1^, Q-factors can be enhanced multiple times in comparison with a single microcavity in the triangular PM, and even can be further improved in other more complicated PM configurations. Therefore, Q-factors are inherently improved in our PM spectrometer. Lastly, the presence of coupled-resonator-induced transparency (CRIT), which is analogous to electromagnetically induced transparency (EIT) in an atomic system and Fano-type asymmetric resonance peaks (see Supplementary Information S11), exhibits a wide distribution across the spectra. These phenomena serve to increase the trapping time of photons within the cavities, resulting in the sharpening of resonance peaks and enhanced diversity within the response matrix.

| Peak serial number | 1 | 2 | 3 | 4 | 5 | 6 | 7 | 8 | 9 | 10 | 11 | 12 | 13 | 14 |
| --- | --- | --- | --- | --- | --- | --- | --- | --- | --- | --- | --- | --- | --- | --- |
| Bandwidth (pm) | 5.6 | 8.1 | 5.7 | 11.2 | 11.1 | 5.5 | 12.6 | 6.9 | 10.8 | 4.9 | 3.5 | 3.1 | 2.0 | 3.0 |
| Loaded Q (×10^5^) | 2.75 | 1.90 | 2.71 | 1.38 | 1.39 | 2.81 | 1.23 | 2.24 | 1.43 | 3.16 | 4.42 | 4.99 | 7.74 | 5.16 |

**Table S1.**  **Bandwidth and Q-factors of the resonance peaks in the measured transmission spectrum.**

**S3. Coupling efficiency diversity**

Due to differing degrees of phase matching and distances of mode distributions from the edge of the microdisk, any single WGM can exhibit unique coupling efficiencies and splitting strengths when interfering with other WGMs, as well as the bus waveguide. This results in each MO exhibiting different wavelength dependences and resonant peak profiles. We will take the heteronuclear diatomic PM as an example to examine the distinct coupling efficiencies of each WGM in different cavities. Fig. **S2a** displays the simulated coupling efficiencies as a function of wavelength, namely $k_{\mathrm{TE}_{0},\mathrm{PA}_{1}}～k_{\mathrm{TE}_{2},\mathrm{PA}_{1}}$, and $k_{\mathrm{TE}_{0},\mathrm{PA}_{2}}～k_{\mathrm{TE}_{2},\mathrm{PA}_{2}}$, which correspond to the coupling from TE_0_ mode of the bus waveguide to TE_0_~ TE_2_ mode of PA_1_ and PA_2_, respectively. The inter-cavity coupling coefficients between TE_0_ ~ TE_2_ of PA_1_ and TE_0_ ~ TE_2_ of PA_2_ are also extracted and visualized in Fig. **S2b**, labeled as $\mu_{\mathrm{TE}_{m},\mathrm{PA}_{1}\&\mathrm{TE}_{n},\mathrm{PA}_{2}}$ where the subscripts *m* and *n* denote the orders of WGMs from 0 to 2. These coupling coefficients are obtained by utilizing a three-dimensional finite-difference time-domain (3D-FDTD) simulator in Lumerical software. The coupling coefficients exhibit significant distinction in strength, often reaching magnitudes of tens. Based on the diverse coupling strengths and propagation constants of involved WGMs, the splitting strength of each MO formed from different orders of WGMs experienced different degrees of dispersion over wavelength. In addition, the variation coupling strengths between WGMs and the bus waveguides further contribute to diversity in the spectral shape such as linewidth. In this way, a greater degree of diversity is introduced into the spectral response, with a corresponding weakening of the overall periodicity.


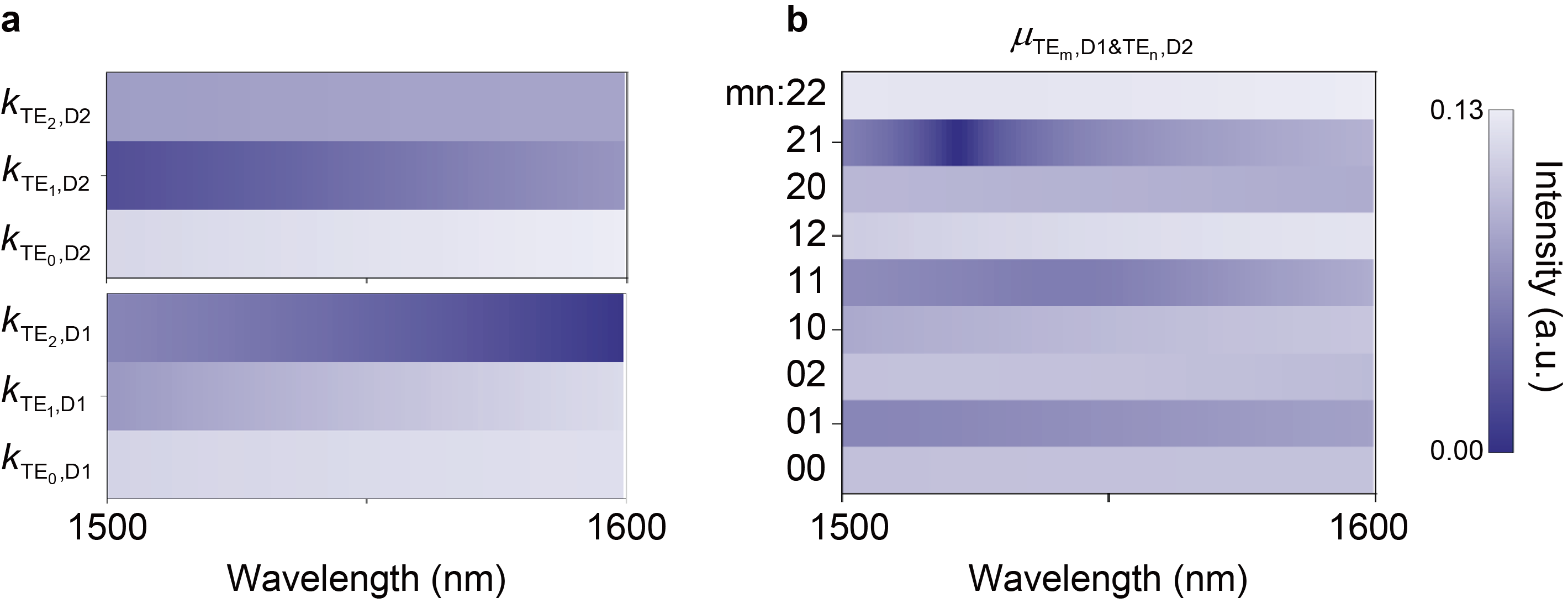


**Fig. S2 | Diversity of coupling efficiencies. a,** Coupling efficiencies as functions of wavelength between bus waveguide and WGMs in PA_1_ and PA_2_. **b,** Inter-cavity coupling efficiencies as functions of wavelength between WGMs in different cavities.

**S4. Field profiles of different super-modes**

For the proposed heteronuclear tetratomic PM, a three-dimensional finite-difference time-domain (3D-FDTD) method is utilized to simulate the spectral response from the drop port, as depicted in Fig. **S3a**.We also monitor electric-field distributions of super-modes that correspond to resonance peaks in different wavelengths as marked with circles and squares with different colors. Electric-field distributions in these super-modes as shown in Fig. **S3b** exhibit strong diversity. Complex super-modes in the PM systems indicate complicated spectral response and imply weakened periodicity.


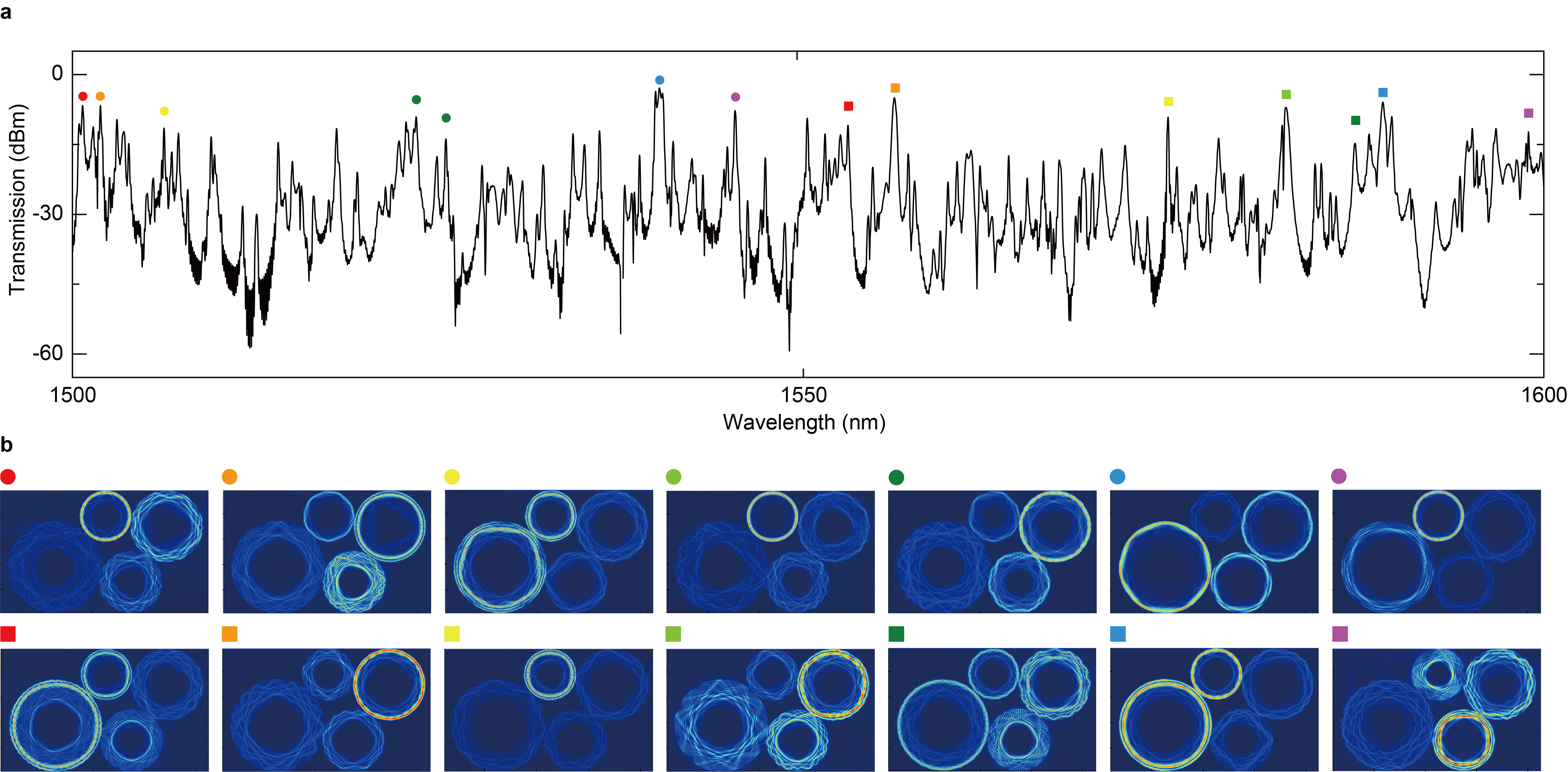


**Fig. S3 |** **Diversity of resonant modes.** Electric-field distribution calculated at the wavelengths corresponding to different super-modes marked with different colors. It can be observed that resonance peaks across the operation bandwidth appear non-periodically because of numerous WGMs in different PAs interfering and interacting with each other.

**S5. Homonuclear and heteronuclear PMs**

Reckoning on the large amount of calculation involved, we employ 3D-FDTD to simulate the spectral response of heteronuclear PM with different radii and homonuclear PMs for comparison, consisting of PAs with a number ranging from one to five. Simulated spectral responses in output ports of PMs are demonstrated in the first and third row of Fig. **S4**, where the orange background refers to the transmission of heteronuclear PM composed of PAs with different radii and the blue background refers to homonuclear PM composed of identical PAs. Obviously, with an increase in the number of photonic atoms, plenty of super-modes are produced in a quasi-random configuration in heteronuclear PMs. The spectral response indicates a heightened level of diversity and produces increasingly significant fluctuations, with a tendency to perform nonperiodic and chaotic behavior. Auto-correlation function is used to evaluate the decorrelation degree and hidden periodicity in the PMs above, which are plotted in the second and last row in Fig. **S4**. Moreover, a more rapid decrease in the auto-correlation function from a value of "1" to "0" indicates a spectral response that offers sharper and faster variations, thereby enabling higher reconstruction resolution. After falling from the initial “1”, auto-correlation of heteronuclear PM contains solely one microdisk atom subsequently ascends to a value of "0.6" and exhibits a pronounced periodicity. Heteronuclear PM with two and three distinct PAs realize effective suppression of periodic to approximately 0.3. and 0.24. Auto-correlation of the heteronuclear PM with four and five distinct PAs announces a rapid fall from value “1” and retains a near value “0” regime. This periodicity-released auto-correlation function indicates a disordered and chaotic spectral response with high diversity, large variation density, imperceptible periodicity, and high reconstruction resolution capability. For comparison, an increase of PA number in homonuclear PM has almost no suppression on the periodicity of the response spectrum, because of the insensitivity of wavelength of MOs formation and insufficient lifting of mode degeneracy owing to the PA configuration with a certain spatial symmetry.


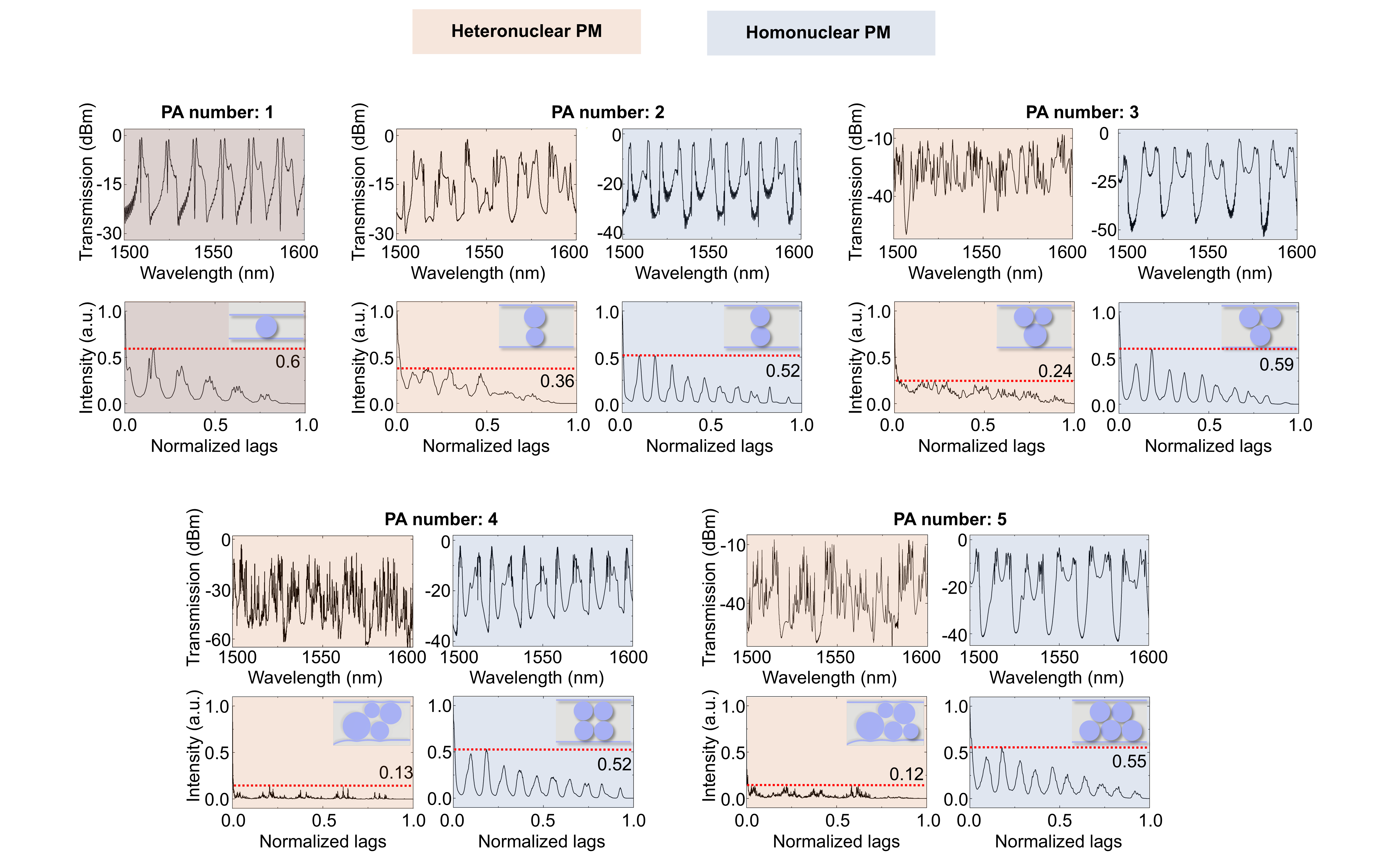


**Fig. S4 |** **Transmissions and auto-correlation for homo- and hetero-PMs with different numbers of PAs.** First row: Simulated transmission spectra of PM consisting of different numbers of PAs; Second row: Calculated normalized auto-correlation functions of transmission spectra in the first row. Orange backgrounds refer to heteronuclear PMs and blue backgrounds refer to homonuclear PMs.

**S6. Systematic investigation of PM configuration and coupling gaps**

Firstly, we investigate the impact of different PM configurations with the same microdisk area. As demonstrated in the manuscript, heteronuclear PMs result in wavelength-dependent splitting strengths of super-modes and complete removal of mode degeneracy due to the breaking of spatial symmetry. This is the critical mechanism behind the quasi-random transmissions characterized by disordered behaviors. To substantiate our findings, we analyzed PMs in five different configurations, each comprising varying combinations of microdisk radii but maintaining the same total microdisk area and coupling gaps. We employed FDTD method to simulate the transmission spectra of these configurations. The results, including the configuration details, simulated transmission spectra, and the calculated auto-correlation function, are presented in Fig. **S5**. Our simulations confirm that all configurations produce diverse and disordered transmission patterns with numerous randomly generated supermodes. Moreover, the periodicity, as quantified by the auto-correlation function, is significantly reduced in all configurations to less than 0.2. Hence, the key to suppressing periodic patterns and creating quasi-random sequences lies in differentiating the size of each microdisk atom to completely eliminate mode degeneracy. Adhering to this principle ensures that various combinations of radii in the same area yield nearly identical effects.


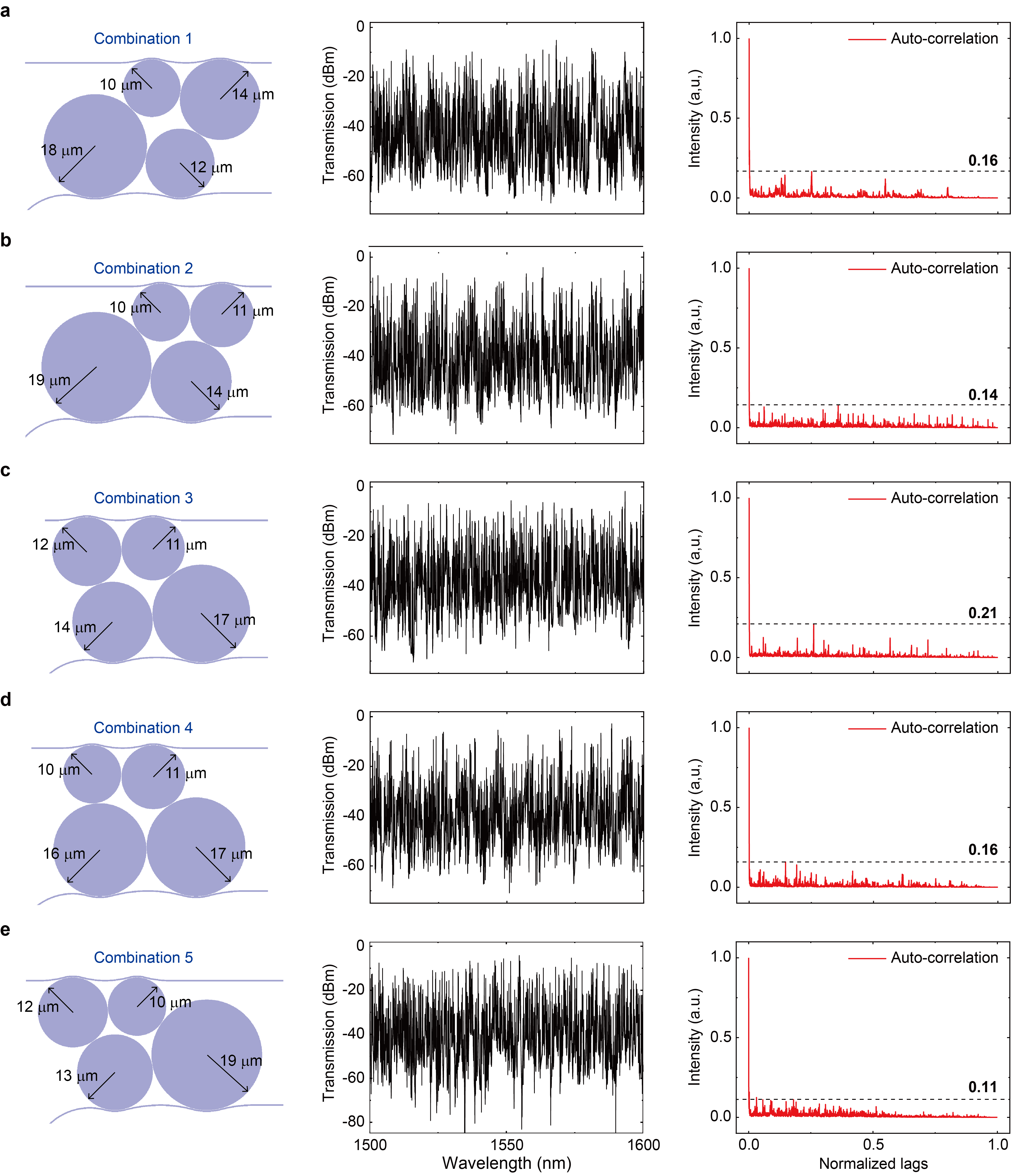


**Fig. S5 |** **Transmission spectra and calculated auto-correlation functions of PMs with five different configurations maintaining identical coupling gaps and total area of microdisks.**

Then, we investigate the impact of coupling gaps on spectrometer performance. For each supermode, the practical Q-factor is co-determined by two components as $Q_{load}=Q_{\mathrm{int}}^{-1}+Q_{\mathrm{ext}}^{-1}$, where *Q*_int_ depends only on the intrinsic loss and *Q*_ext_ signifies the Q-factor reduction due to the external power loss caused by evanescent coupling to the bus waveguides. FWHMs, determined by *Q*_load_, reflect the degree of transmission sharpness and are the decisive factor of reconstruction resolution. Transmission spectra of a series of PMs with identical configurations but varying coupling gaps are simulated. These coupling gaps between microdisks, as well as between microdisks and bus waveguides are uniformly adjusted from 120 nm to 220 nm. The simulated transmissions and calculated auto-correlation functions are exhibited in Fig. **S6**. The variation of coupling gaps has slight perturbation on the periodicity suppression effect and randomness of the transmission. The estimated reconstruction resolutions, characterized by Eqn.6 in the manuscript, are illustrated in Fig. **S7**. It is evident that as the coupling gap widens to 220 nm, the resolution is improved from 74 pm to 4 pm.


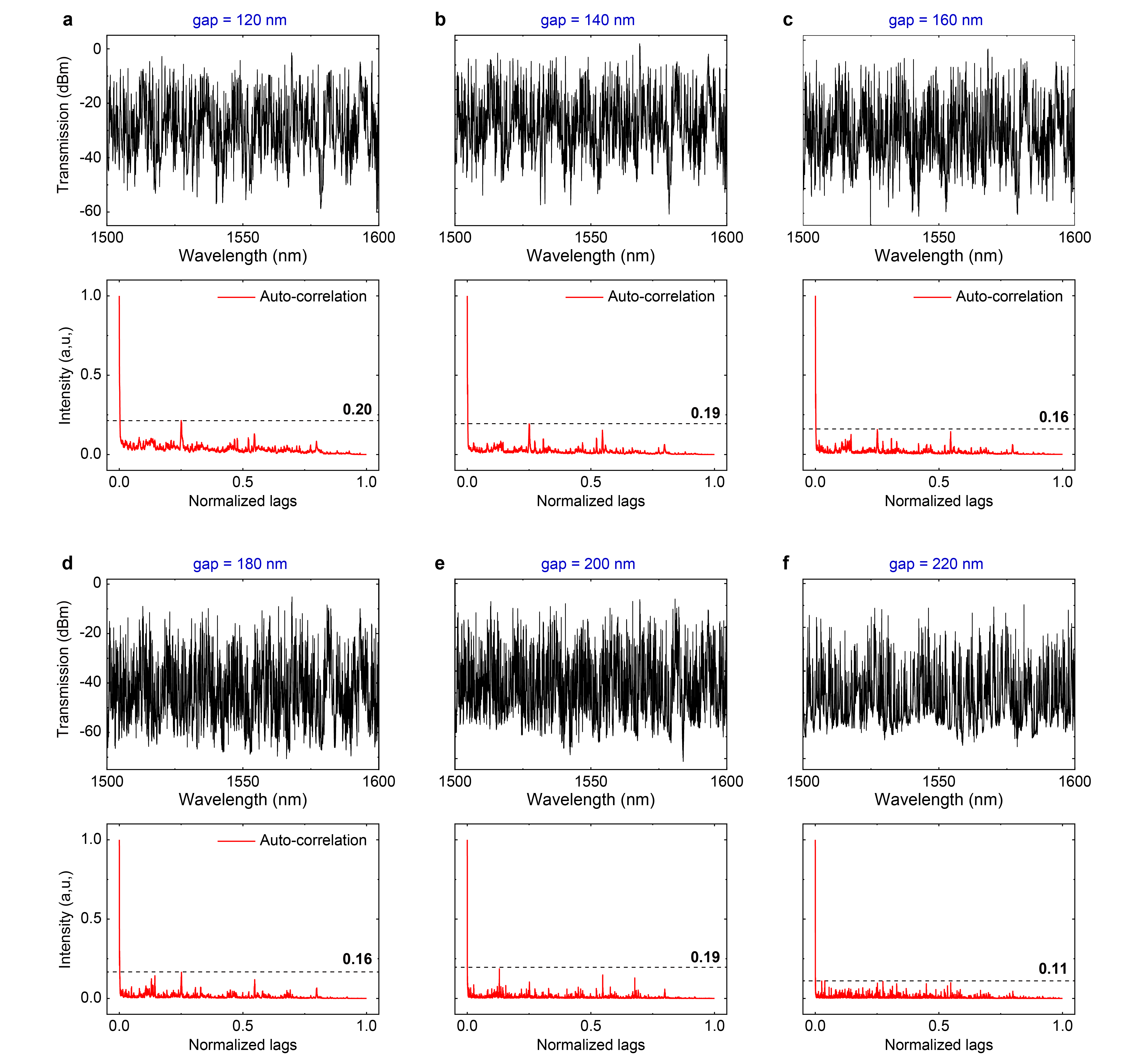


**Fig. S6 |** **Transmission spectra and calculated auto-correlation functions of PMs with varying coupling gaps of** **a,** 120 nm; **b,** 140 nm; **c,** 160 nm; **d,** 180 nm; **e,** 200 nm; **f,** 220 nm; maintaining the same configurations.


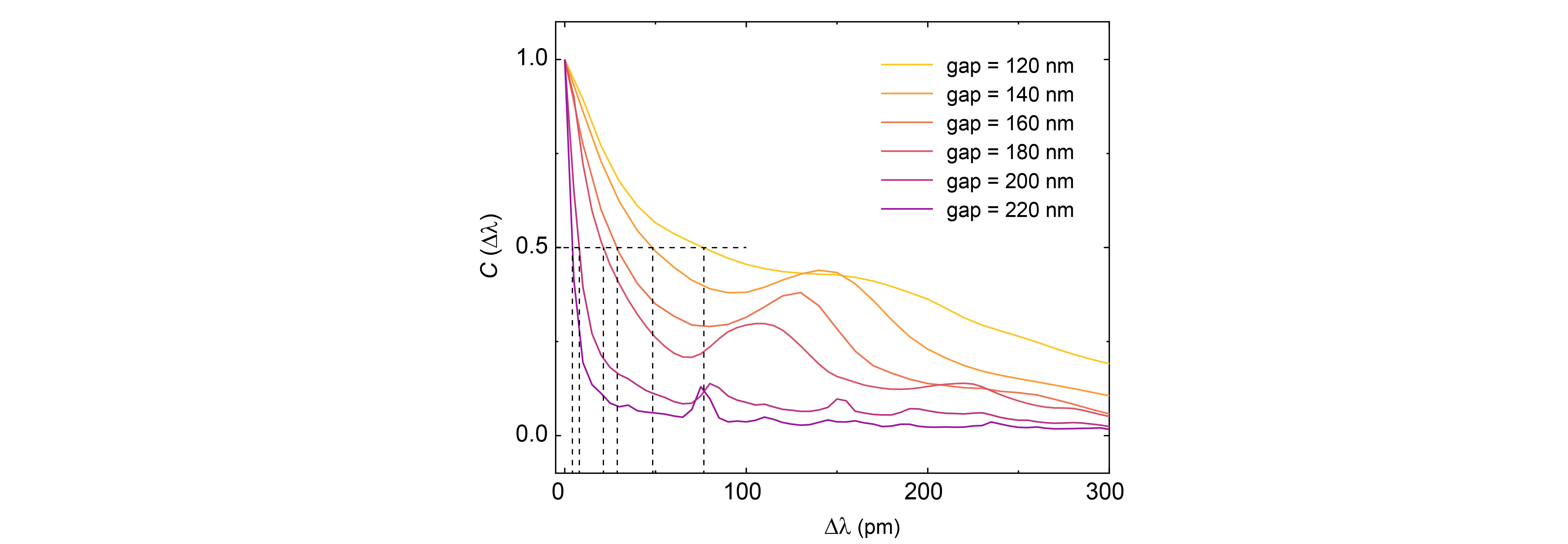


**Fig. S7 | Estimated reconstruction resolution of PMs with varying coupling gaps.** Black dashed line depicts the estimated resolution. $C(\Delta\lambda)$ represents the spectral correlation function of spectral response, $\Delta\lambda$ is the spectral spacing between two distinct wavelengths in spectrum.

Lastly, we want to emphasize that, randomness is the cornerstone of compressive sensing and computational spectroscopy based on CS, and is the core guideline to motivate the development of computational spectrometers. Unlike traditional optical systems that seek to eliminate disorder, computational spectrometers intentionally introduce randomness and diversity. Driven by the core concept of randomness, our proposed approach seeks to demonstrate a design criterion with a high tolerance for parameters. The dimensions of the microdisks, including variables like the gap and combinations of microdisk radii, are chosen randomly, devoid of specific constraints. Combining the above discussion, the analysis of PA numbers in the main text, and the experimental measurements of microdisk region and coupling efficiency, the final structure of the PM is finally determined. Given the inherent FSR limitation in resonant devices, a greater demand for randomness and disorder arises. This approach results in coupling coefficients and multimode interference characterized by significant diversity, addressing the inherent free spectral range (FSR) limitations in resonant devices.

**S7. Response matrix redundancy in PMs with different numbers of PAs**

The singular-value decomposition (SVD) approach is employed to analyze the response function provided by heteronuclear PM, which comprises single, double, and tetra microdisk atoms, respectively. Firstly the spectral transmissions of these three types of PMs are reshaped into a matrix with a dimension of *k*×*l*. Here, *k* = 20 indicates that the one-dimensional transmission is divided into *k* sections to investigate the information density within each section. By exploiting SVD, **T**can be uniquely expanded as a factorization of a rectangular diagonal matrix (**Σ**) with non-negative real numbers on the diagonal and two complex unitary matrices (**U** and **V**):

$$\begin{aligned} \mathbf{T}_{k\times l}=\mathbf{U}_{k\times k}{\boldsymbol{\Sigma}_{k\times l}\mathbf{V}}_{l\times l}^{*}\#\left( S5 \right) \end{aligned}$$

Where **U** and **V** are left- and right-singular vectors of **T**. The diagonal entries $\sigma_{i}=\boldsymbol{\Sigma}_{ii}$ denote the singular value of **T**, formed in descending order. Typically, the valid information is observed to primarily concentrate on the first few singular values. The rate of descending in singular values indicates the level of information redundancy. Thus, $\sigma_{i}/\sigma_{max}$ as a function of *i*for three types of PMs are plotted in Fig. **S8** to evaluate the quality of spectral transmission. As the quantity of PA increases, the rate of decline of the singular value $\sigma_{i}$ decreases significantly, proving a stronger decorrelation degree of the information contained in each row of **T**. The proposed heteronuclear tetra PM delivered high content valid information throughout the entire operation bandwidth, ensuring high encoding efficiency and reconstruction resolution via the de-periodic spectral response matrix.


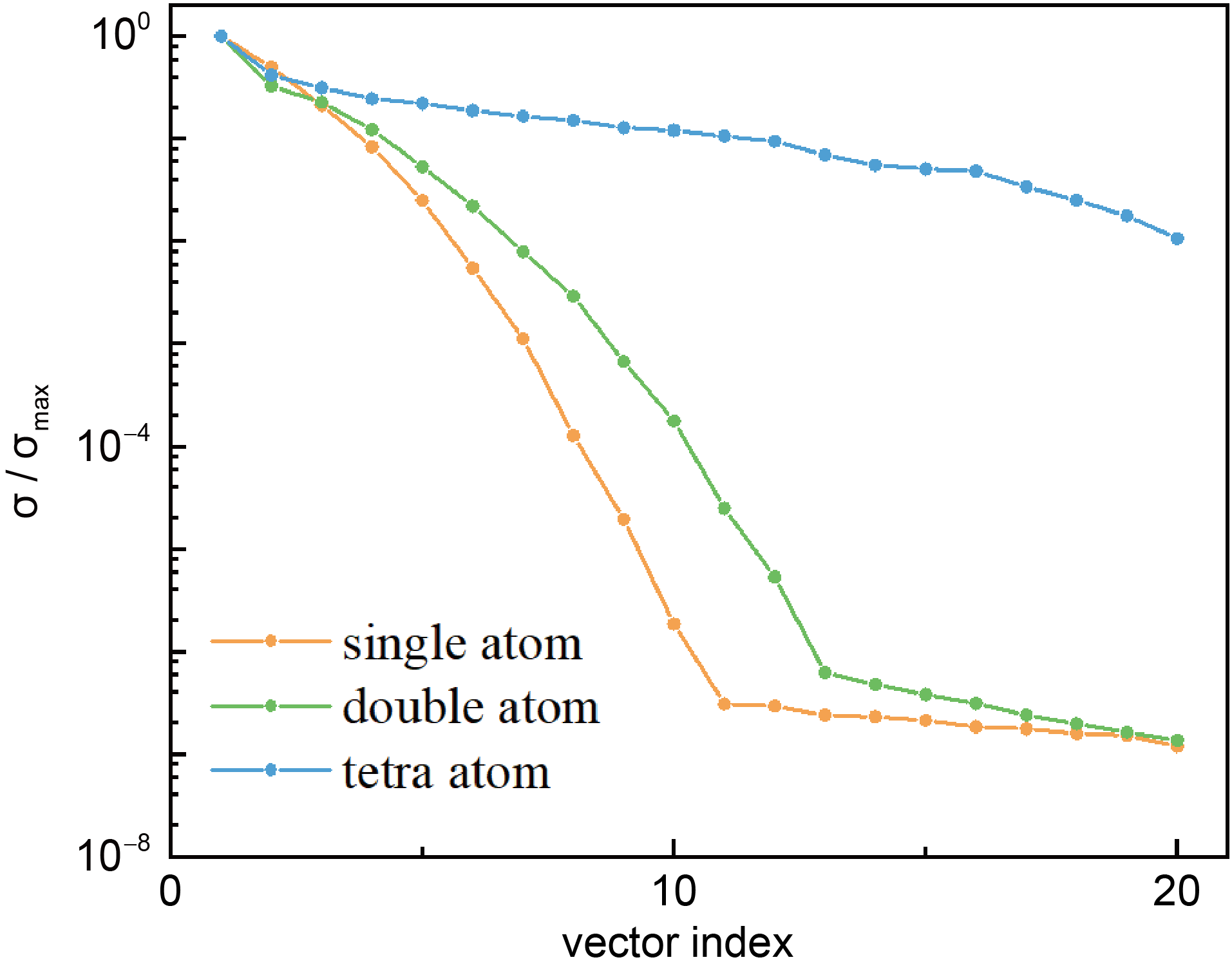


**Fig. S8 |** $\sigma_{i}/\sigma_{max}$ **of SVD processed transmission spectra as functions of vector index *i* for monatomic PM, diatomic PM, and tetratomic PM, respectively.**

**S8. Optical microscopy photos of microdisk regions.**

Optical microscopy photos of functional microdisk regions with and without TiN heaters are provided in Fig. **S9**.


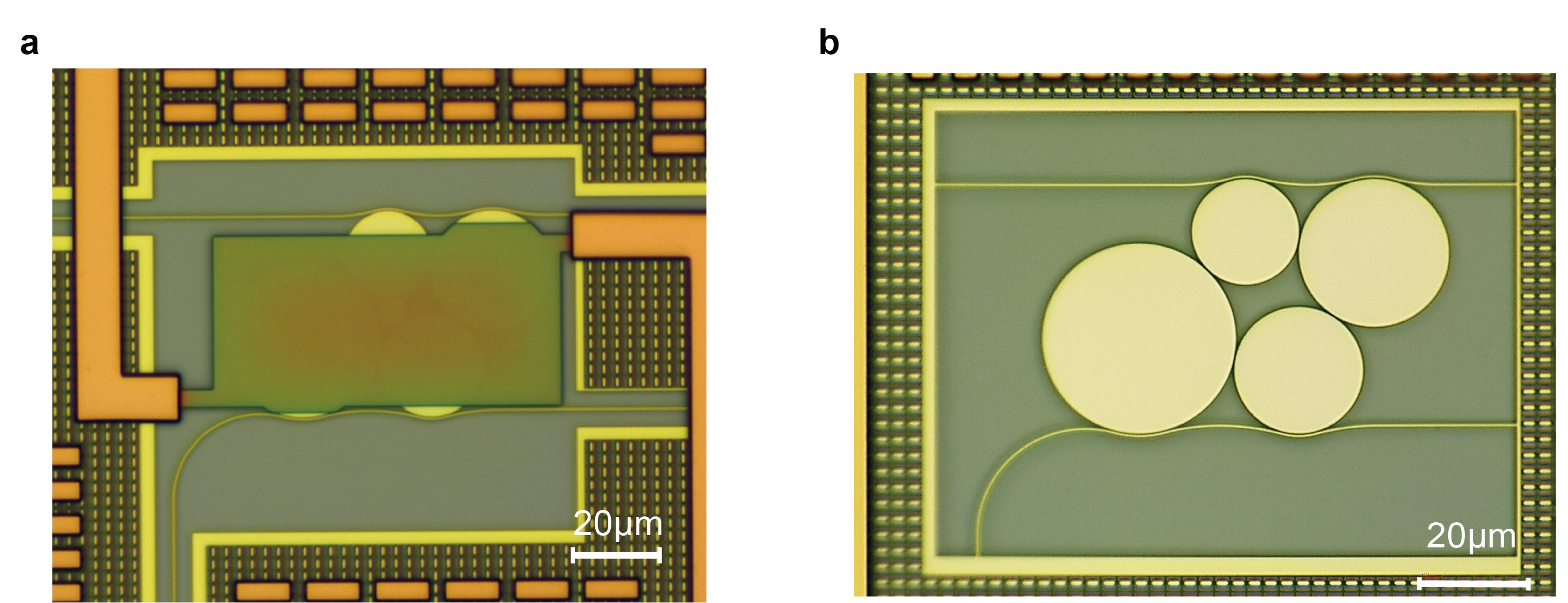


**Fig. S9 | Optical microscopy photos**. Microdisk regions with **a,** and without TiN heater **b**.

**S9. Heating principle and role.**

Heating pads and metal routing lines are deposited by 850 nm thick copper, with dimensions of 60×80 μm^2^ and width of 10 μm. The titanium nitride layer is deposited via the Atom Layer Deposition system (ALD). The resistances of the copper metal layer and TiN heater are 25 $m\Omega\mathrm{sq}^{-1}$and 15 $\Omega\mathrm{sq}^{-1}$, respectively. These two layers are interconnected by through silicon vias (TSV) measuring 450 nm in thickness. The TiN heater layer is positioned 850 nm above the silicon waveguide layer. Therefore, applying external voltages to the heating pads enables heating of both the interconnected TiN heater later and the underneath silicon waveguide layer. Silicon has a positive thermos-optic coefficient ($dn(\mathrm{Si})/dT=1.86\times{10}^{-4}K^{-1}$ around 1550 nm) and its refractive index increases with raised temperature. For each whispering gallery mode in the PM spectrometer, the resonance wavelength can be calculated as:

$$\begin{aligned} \lambda_{\mathrm{res}}=n_{\mathrm{eff}}\cdot\frac{L}{m}, m=1,2,3\ldots\#\left( S6 \right) \end{aligned}$$

Thus, the increased temperature by external voltages induces red-shifting in resonance wavelengths of each mode.

A truncated response matrix with a wavelength range from 1540 nm to 1550 nm is plotted in Fig. **S10a**. Transmission spectra under 1^st^, 150^th^, and 300^th^ heating powers are plotted in Fig. **S10b**. In our design, the heater covers nearly all the microdisk regions, aiming to stimulate all supported resonant modes to induce wavelength shifting under the elevated temperature through the TO effect. Each row in the response matrix comprises transmission spectra under different heating powers. In general, TO effect is utilized to generalize different patterns of transmission to construct the response matrix to encode and sample incident signals.


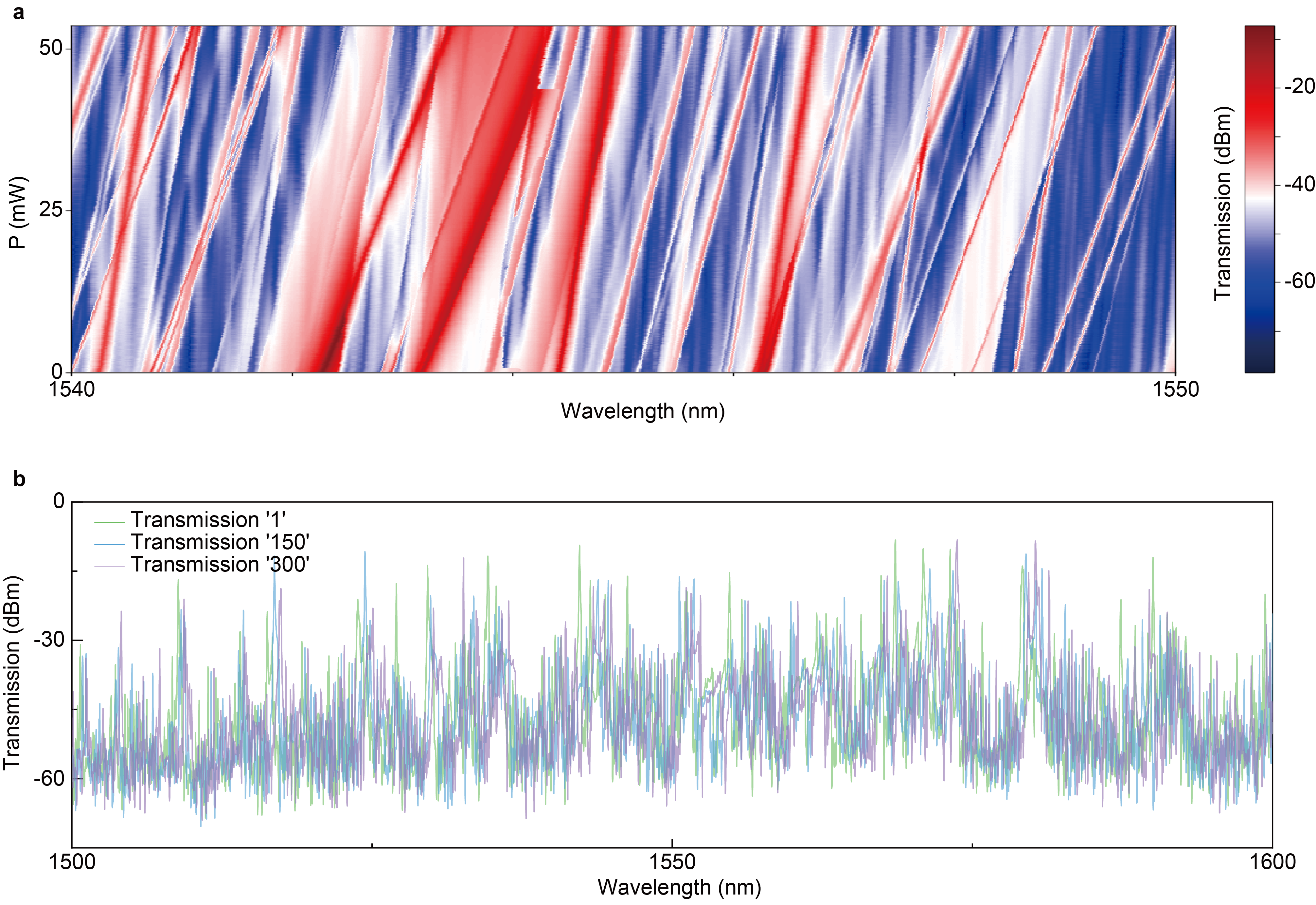


**Fig. S10 | a, Truncated measured response matrix from 1540 nm to 1550 nm. b, Transmission spectra under 1^st^, 150^th^ and 300^th^ heating channels.**

Based on our disordered PM spectrometer, applying larger external voltages leads to greater wavelength shifting of each supermode and amplifies mode interference and hybridization effects. This contributes to the decorrelation of each column in the response matrix, thereby improving orthogonality and robustness against measurement noise. Yet, unstable electrical current fluctuations are observed when the external bias exceeds certain thresholds, thus the external powers applied to the heating pads demonstrated in the manuscript are experimentally pre-selected to ensure stable operation.

**S10. Tuning efficiency**

Due to the distinct mode distributions of different supermodes, each exhibiting varying thermos-optic sensitivity, we summarize the tuning efficiency of supermodes with respect to wavelength as plotted in Fig. **S11**. The overall tuning efficiency is estimated to be 15.24 $pm mW^{-1}$.


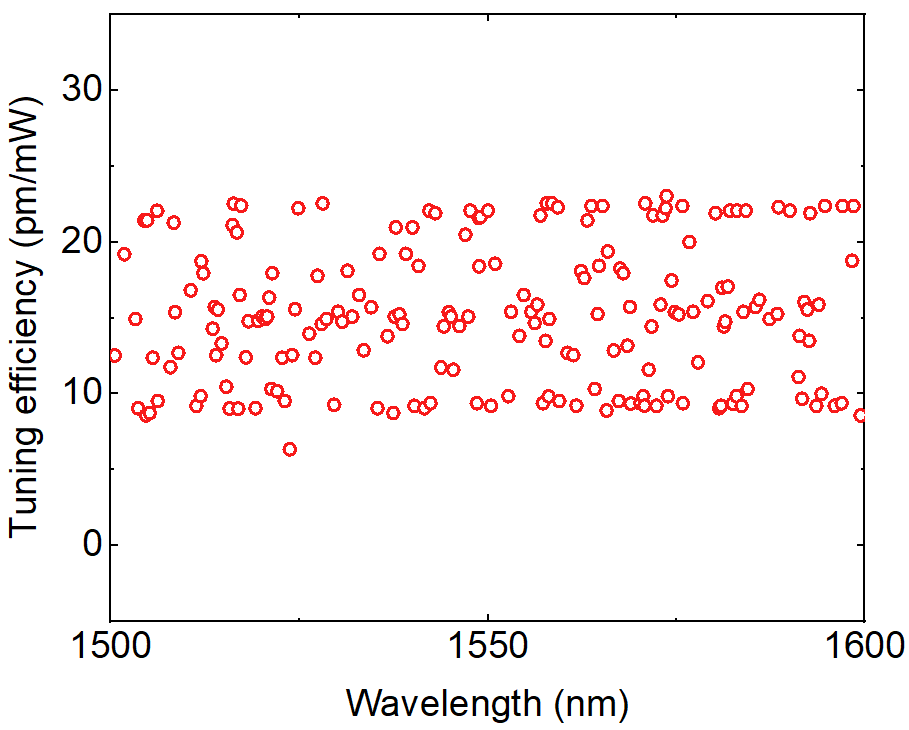


**Fig. S11 | Tuning efficiencies of supermodes with respect to wavelength.**

**S11. CRIT, Fano, and mode evolution effects**

In the response matrix of the proposed tetratomic PM spectrometer, some coherent interference effects such as coupled-resonator-induced transparency (CRIT) which can be analogous to electromagnetically induced transparency (EIT) in atom system, and the Fano effect are frequently witnessed across the whole operation bandwidth. Consider any two WGMs in different microdisk atoms in on-resonance conditions. The matched resonance frequency of the two WGMs results in the emergence of a narrow transparent window on the bandpass-type resonance peak, which is CRIT, as a result of destructive interference between the two WGMs. This CRIT phenomenon can be observed experimentally as depicted in Fig. **S12a**, as labeled by red dashed boxes. When the resonance frequencies of two WGMS are detuned, sharp asymmetric spectra appear similar to the Fano-type resonance shape, which is identified by blue dotted boxes. The presence of CRIT and Fano-type resonances with varying features can be widely observed across the entire spectrum of responses, which significantly increases spectrum fertility and randomness and further liberates the inherent periodicity restriction of WGM spectral response in individual microdisk cavities.

Furthermore, we consider the response matrix under linearly swept bias. In our design, the heater covers almost all the microdisk region to drive all supported resonant modes to generate wavelength shifting under the raised temperature via the TO effect. Different WGMs yield different temperature sensitivity, thus mutual coupling can be manipulated. As depicted in Fig. **S12b**, individual WGM gradually split into two or three super-modes along with the increased power channel in ⅰ and ⅲ, and canceled resonance can also be recovered with the varied heating power, as depicted in ⅱ. Numerous super-modes experienced different interferences and interactions under different power channels, which go beyond pure resonance wavelength red-shifting.


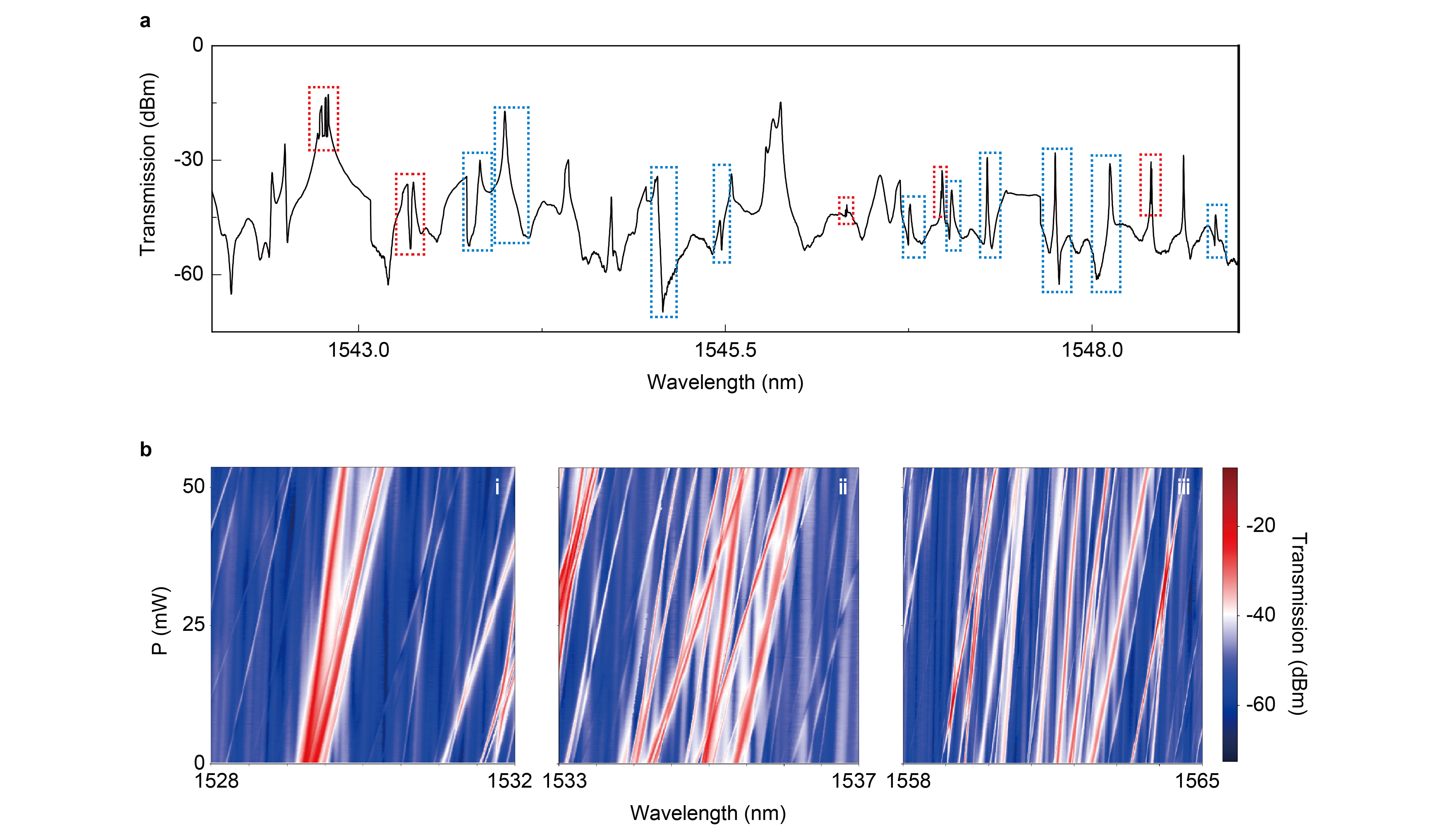


**Fig. S12 |** **a,** The intercepted segment of the initial transmission spectrum. Red dashed boxes label the CRIT phenomenon while blue dashed boxes label asymmetric Fano resonance. **b,** The intercepted segment of the response matrix under heating powers.

**S12. Picard condition analysis**

First, spectra of test random and Gaussian signals in Fig. **3c** in the manuscript are shown in Fig. **S13a**. *Picard plots* of square signal and superposition of sin-function signals are plotted in Fig. **S13b** and **c.** We emphasize that it is the decay rate of $\sigma_{i}$ and SVD coefficients $u_{i}^{*}\mathbf{I}$ that matters whether it satisfies the discrete Picard condition. A faster decrease of SVD coefficients than $\sigma_{i}$ for $i\leq50$is observed. This is also can be verified from the initial descending of solution coefficients $u_{i}^{*}\mathbf{I}/\sigma_{i}$ and even in higher vector index, no apparent and harsh increase is witnessed. For the larger vector index, one should ignore the part of the *Picard plot* that corresponds to tiny singular values that $u_{i}^{*}\mathbf{I}$ level off at some noise plateau^2^. For our PM spectrometer, the discrete Picard condition is satisfied for different kinds of archetype signals that stable and convergent solutions can be reached.


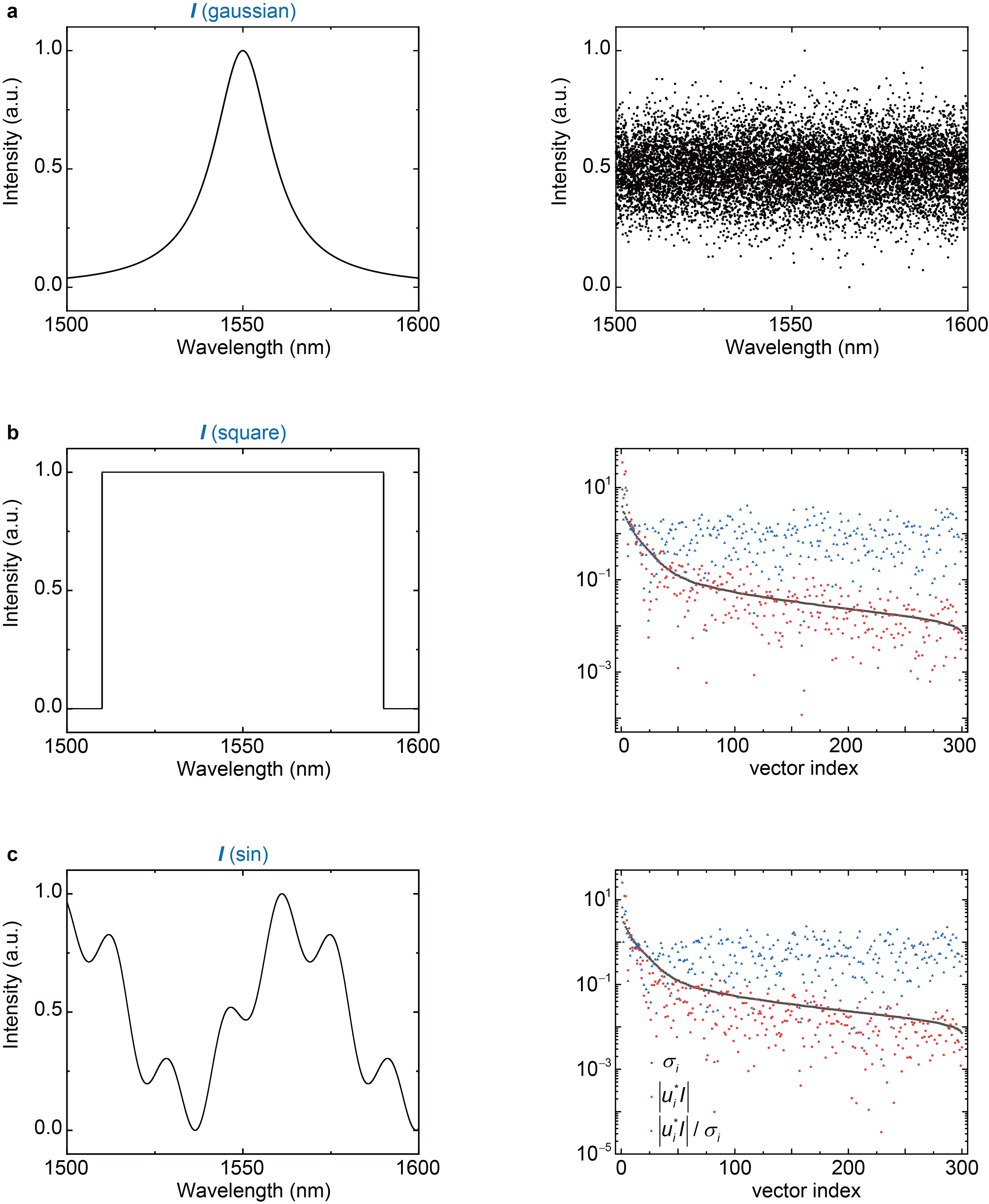


**Fig. S13 |** ***Picard plots*.** **a,** spectra of test signals demonstrated in Fig. **3c** in the manuscript.

**b**, *Picard plots* of square and sine-function spectral features.

**S13. Numerical simulation based on measurement response matrix**

Numerical calculations are performed to assess the efficacy of the proposed spectrometer in retrieving a series of probe signals in three archetypal spectra: discrete (Fig. **S14a**), continuous (Fig. **S14b**), and step signal (Fig. **S14c**). We mathematically generate probe signals of *M_w_* wavelength points as exhibited in the first column of Fig. **S14** with blue backgrounds, which are then fed into the measured response matrix. Calculated optical power in different heating power channels can be calculated as $\mathbf{S}_{N_{p}\times1}=\mathbf{T}_{N_{p}\times M_{w}}\mathbf{I}_{M_{w}\times1}$. We first reconstruct the probe signals by directly solving the under-determined linear algebra function as equation (9). The reconstructed and corresponding relative errors *ε *are calculated and plotted in the middle row of Fig. **S14** with yellow backgrounds. It is noteworthy to mention that the experimentally pre-calibrated response matrix is nearly ill-posed (*N*≪*M*). Obviously, sparse discrete signals can be accurately resolved, while continuous and step probe signals are highly misinterpreted with large relative errors. This issue arises from the inherent limitations of compressive sensing, as continuous signals violate the sparsity principle. Prior information and constraints must be incorporated as regularization in order to compensate the information loss, even in the noise-free numerical simulations. Continuous signals are intrinsically more challenging to address than discrete signals that adhere to the sparsity principle. Details can be found in Supplementary Information S16.

The reconstruction results after proper regularization are presented in the third column of Fig. **S14**. The relative errors are significantly suppressed. We introduce $\alpha_{1}\left\| \mathbf{I} \right\|_{1}$ as regularization term when tacking with discrete signals; Tikhonov regularization that $\alpha_{2}\left\| \mathbf{I} \right\|_{2}+\alpha_{3}\left\| D\mathbf{I} \right\|_{2}$are introduced as regularization terms in continuous signal processing. Total variation (TV) regularization and Tikhonov regularization are utilized simultaneously that $\alpha_{3}\left\| D\mathbf{I} \right\|_{2}+\alpha_{4}\left\| D\mathbf{I} \right\|_{1}$ are introduced to the step signal processing. Total variation (TV) smoothing allows for some steep gradients, while the *l*_2_-norm of the first derivation of **I** does not allow any steep gradients thus producing highly smooth solutions, is therefore able to produce piecewise smooth solutions. Clearly, the response matrix that was experimentally determined is capable of processing different kinds of archetype signals, with the aid of appropriate regularization. Hence, sophisticated incident spectra with a variety of optical features can be precisely distinguished and retrieved based on our proposed encoder if the experimental environment is sufficiently stable.


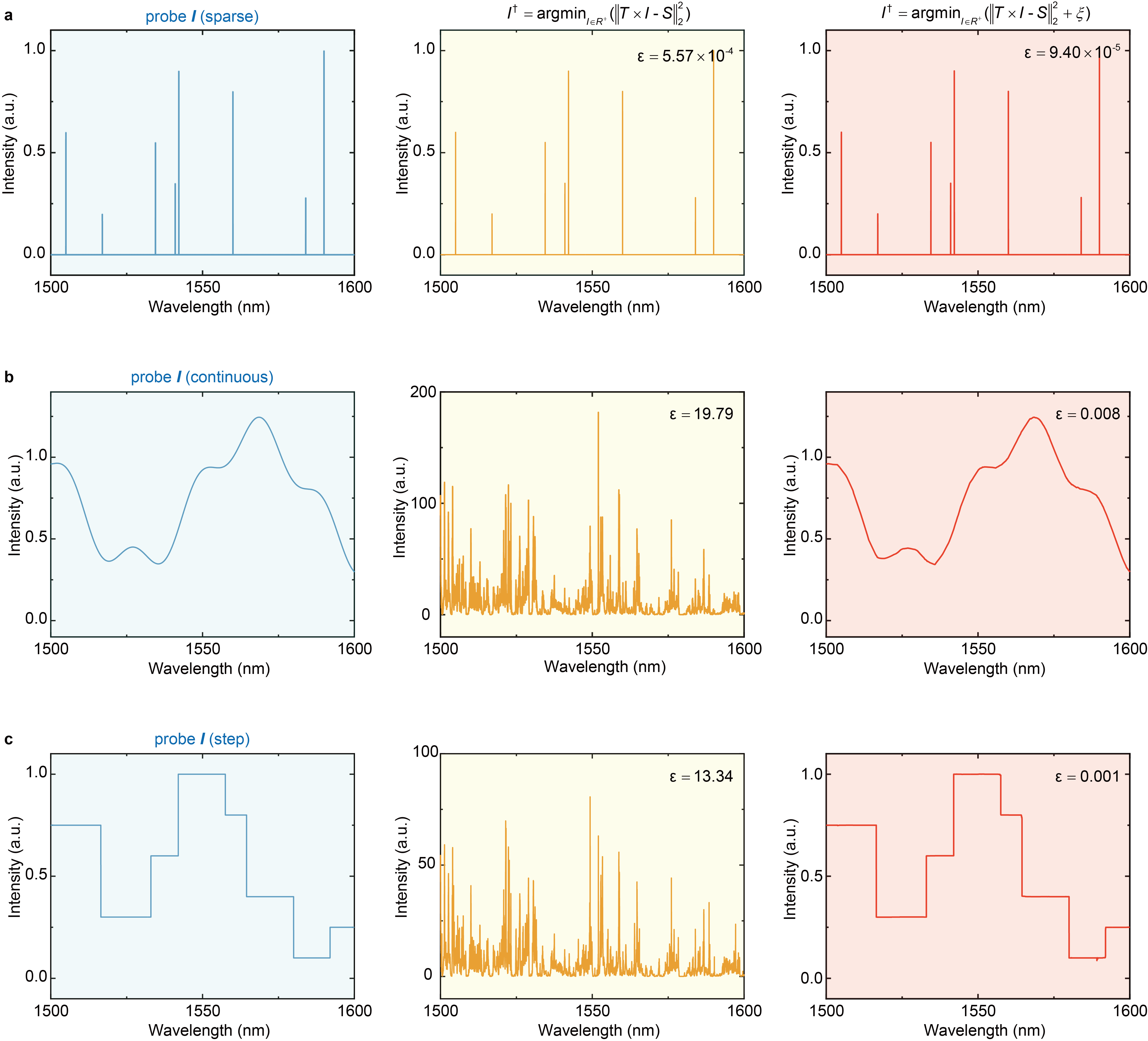


**Fig. S14 |** **Numerical reconstruction**. Test spectra are with **a**: discrete, **b**: continuous, and **c**: step features. First column with blue backgrounds: test input spectra. Second and third columns with yellow and red backgrounds respectively: Reconstruction results with and without regularization coefficients, where ξ refers to appropriate regularization.

**S14. Signal-to-noise ratio**

We further investigate the SNR under a narrower operational bandwidth of 20 nm, which signifies an ill-conditioned inverse problem with dimensions of 300×2000. Our disordered PM spectrometer exhibits an SNR exceeding 22 dB in 20 nm bandwidth. SNR tests are illustrated in Fig. **S15**, with reconstruction accuracies and SNR values labeled.


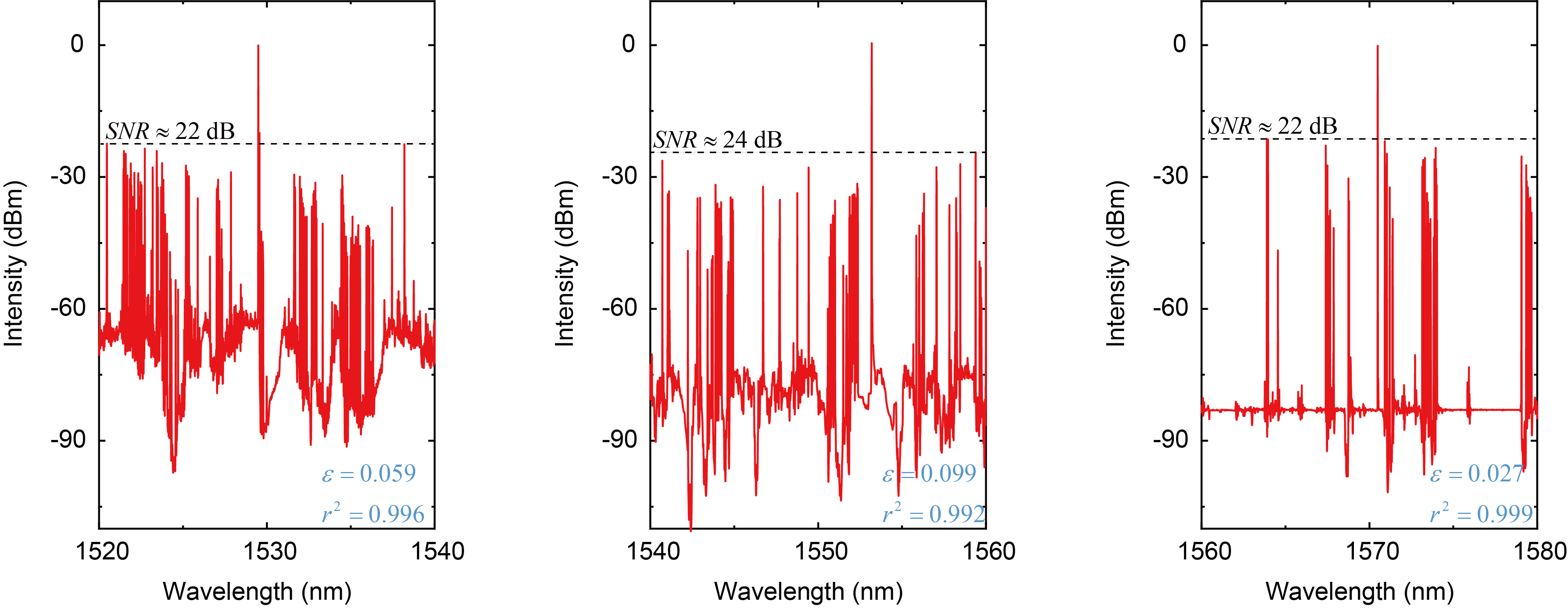


**Fig. S15 |** **SNR test** under 20 nm operational bandwidths. Black dashed lines labeled noise level. *ε* and *r^2^* are labeled.

**S15. Representative continuous signals reconstruction**

Additionally, we have generated band-pass signals and Gaussian signals, which are prevalent and emblematic in communication systems, using a broadband ASE laser source and a waveshaper. The reconstructed signals, along with benchmarks measured by a commercial OSA, are illustrated in Fig. **S16**.


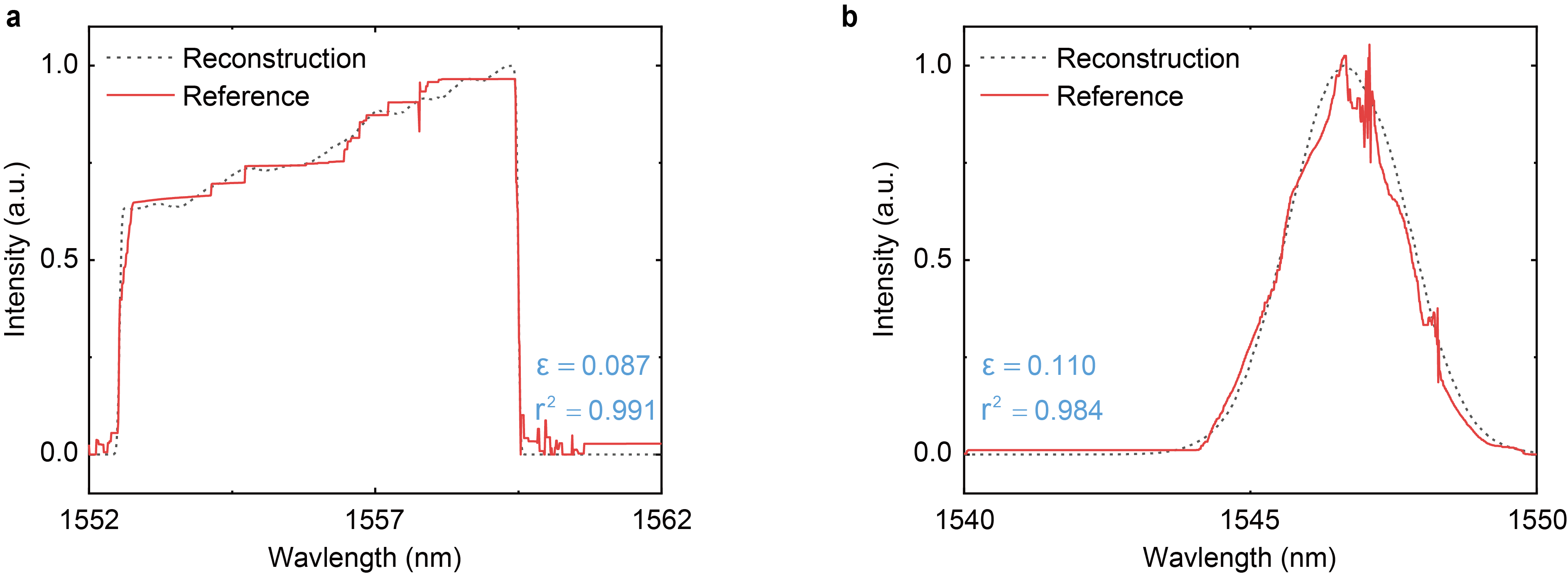


**Fig. S16 |** **Reconstruction results of real-world band-pass signal and Gaussian signal.**

**S16. Analysis of continuous signals reconstruction quality**

The factors influencing the accuracy of measured continuous spectra from the following perspectives.

1. In our disordered PM spectrometer, compressive sensing is employed. The dimension of the response matrix is of 300×10^4^. A core principle of compressive sensing is sparsity. This refers to the property where a signal is represented by a sparse set of coefficients in a transform domain, with the majority being zero or nearly zero. This allows for significant dimension reduction without losing information content, facilitating efficient signal reconstruction with fewer sampling channels and a smaller measurement matrix, and less reliance on prior knowledge. However, challenges arise when compressive sensing is applied to continuous signals that are not inherently sparse and contain many non-zero coefficients, leading to information loss, especially under high compression ratio conditions. To address this, one might consider increasing more sampling channels, or incorporating more prior information and constraints, which necessitates more sophisticated algorithms and complicates parameter tuning. This is the intrinsic reason why continuous signals are more difficult to recover compared to discrete signals using reconstruction algorithms. This can be validated by numerical calculation simulation presented in Supplementary Information **S13**. Without appropriate regularization, discrete signals can be resolved with greater accuracy minimal error. However, the response matrix become incapable of resolving smooth and stepped continuous uncapable to solve continuous signals, which are completely misinterpreted. Appropriate regularization is crucial when solving continuous signals, while this requirement can be bypassed for discrete signals because of sparsity principle.
2. Consider discretization process. Our disordered PM spectrometer, characterized by widely distributed high-Q resonant peaks, exhibits significant variations in real transmission around the resonant wavelengths. These variations are not captured when sampling the response matrix with a wavelength grid equal to or larger than the peak bandwidth. For instance, when sweeping and sampling with a wavelength grid of 1 pm, the bandwidths of some resonance peaks, as detailed in Table. S1 in Supplementary Information S2, are smaller than 10 pm, which can be recorded and observed. However, when sampled using a coarser wavelength grid of 10 pm, these peaks become distorted or even disappear entirely. Transmission spectra of the same disordered PM spectrometer when sampled in 1 pm and 10 pm are exhibited in Fig. **S17**. This discrepancy can lead to large errors when using the pre-calibrated responses sampled with a coarse wavelength grid (like 10 pm) to solve the collected output power **S** transmitted and filtered after the ground-truth spectral responses.

In fact, this drawback is inherently due to the ultra-high Q-factor of the device and becomes more pronounced in the response matrices with sharper optical features and drastic changes, which are essential for achieving high reconstruction resolution. Essentially, there exists a trade-off between spectral resolution and solvability for continuous signals. By adapting a response matrix with smoother features with less fluctuation, while sacrificing spectral resolution, helps to minimize this discretization error and delivers more accurate solutions for continuous signals. This trade-off can be mitigated by increasing the number of sampling points with a finer wavelength grid and increasing sampling channels simultaneously, although this significantly increases computational demands and the complexity of the solution process.


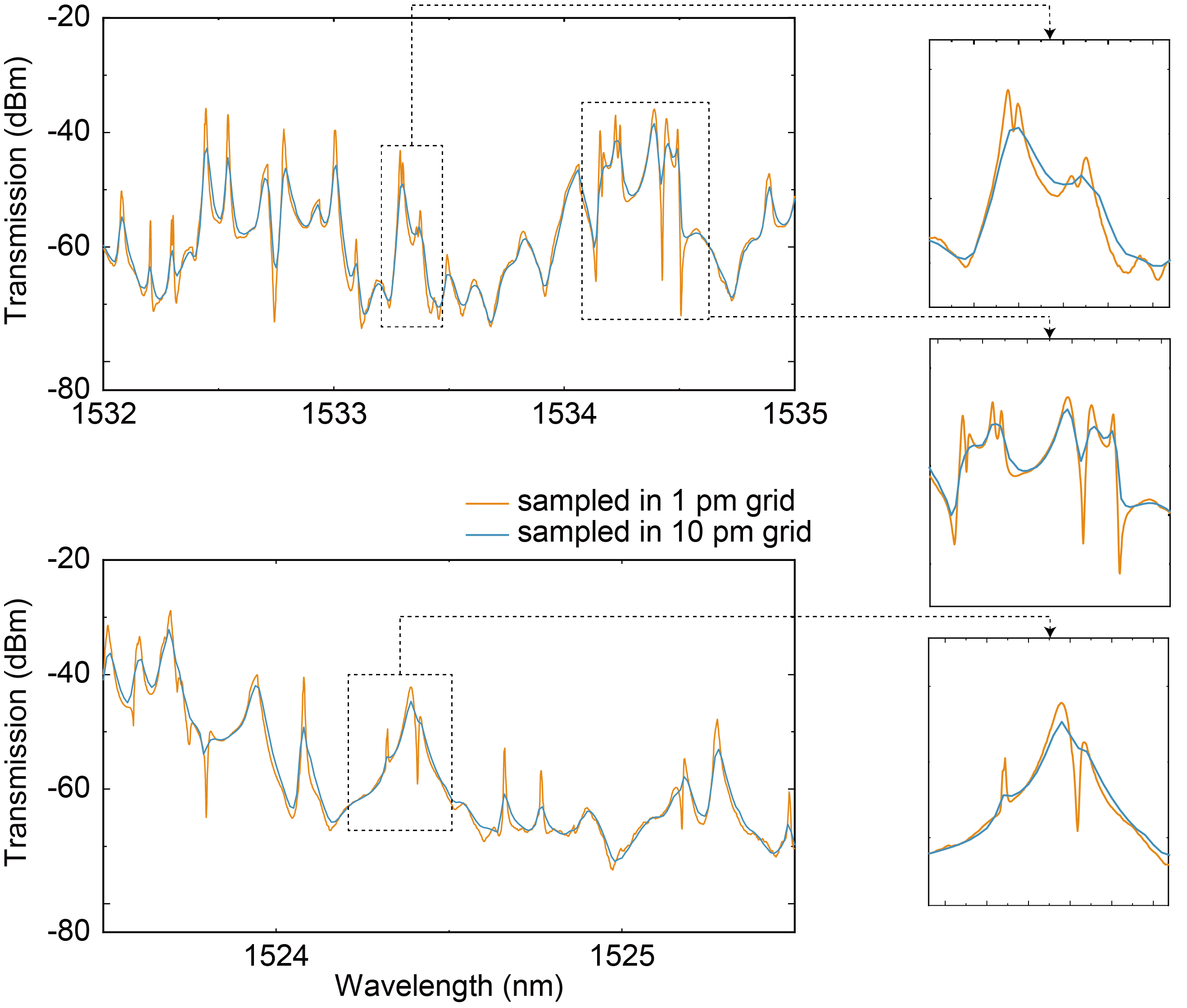


**Fig. S17 | Transmission spectra of the same disordered PM spectrometer when sampled with 1 pm and 10 pm wavelength grid, respectively.** Insets show some zoom-in transmissions that cannot be correctly captured under a coarse wavelength grid, as labeled by black dashed squares.

1. The absence of an active optical bench along with a pneumatic system, and a temperature controller also introduces large measurement noises, as discussed in the main text.

**S17. Measurement results for heteronuclear triatomic PM**

We also tested other heteronuclear PMs that consist of three microdisk atoms with radii of 6 μm, 8 μm, and 11 μm, respectively. The optical microscopy photo of the fabricated triatomic spectrometer device is exhibited in Fig. **S18a**. The insets represent the zoom-in microscopy of microdisk PAs region with and without a heater, respectively, where the function region only with an ultra-compact footprint of 30×35 μm^2^. A max power *P*_max_≈60 mW external power is applied, with $\delta P$ as the sampling step of about 0.3 mW; *BW* is the spectrometer measurable bandwidth that is from 1500 nm to 1600 nm; $\delta\lambda$ is the wavelength grid of 10 pm. Fig. **S18b** exhibits the experimentally calibrated response matrix under different heating power channels and the initial transmission spectrum under *P* = 0 mW is provided in Fig. **S18c**. The estimated resolution based on the response matrix can be numerically calculated as eqn. (6) in the text part, where FWHM is labeled of 80 pm in Fig. **S18d**. Because of the smaller number of PAs and each PA yielding a smaller radius, mode hybridization and splitting strength evolution are weakened in comparison to our optimal device exhibited in the manuscript, hence delivering a compromised resolution. However, with a smoother optical transmission that suppresses the sharpness contained in the response matrix, it can deliver lower error when interpreting continuous incident signals. As illustrated in Fig. **S18e**, where the reference spectra are exhibited in the left, and reconstructed spectra are exhibited in the right figure with a relative error *ε*=0.108 and coefficient of determination *r*^2^=0.988. The reconstruction accuracy for continuous signals is effectively improved in comparison with the spectrometer demonstrated in the text.


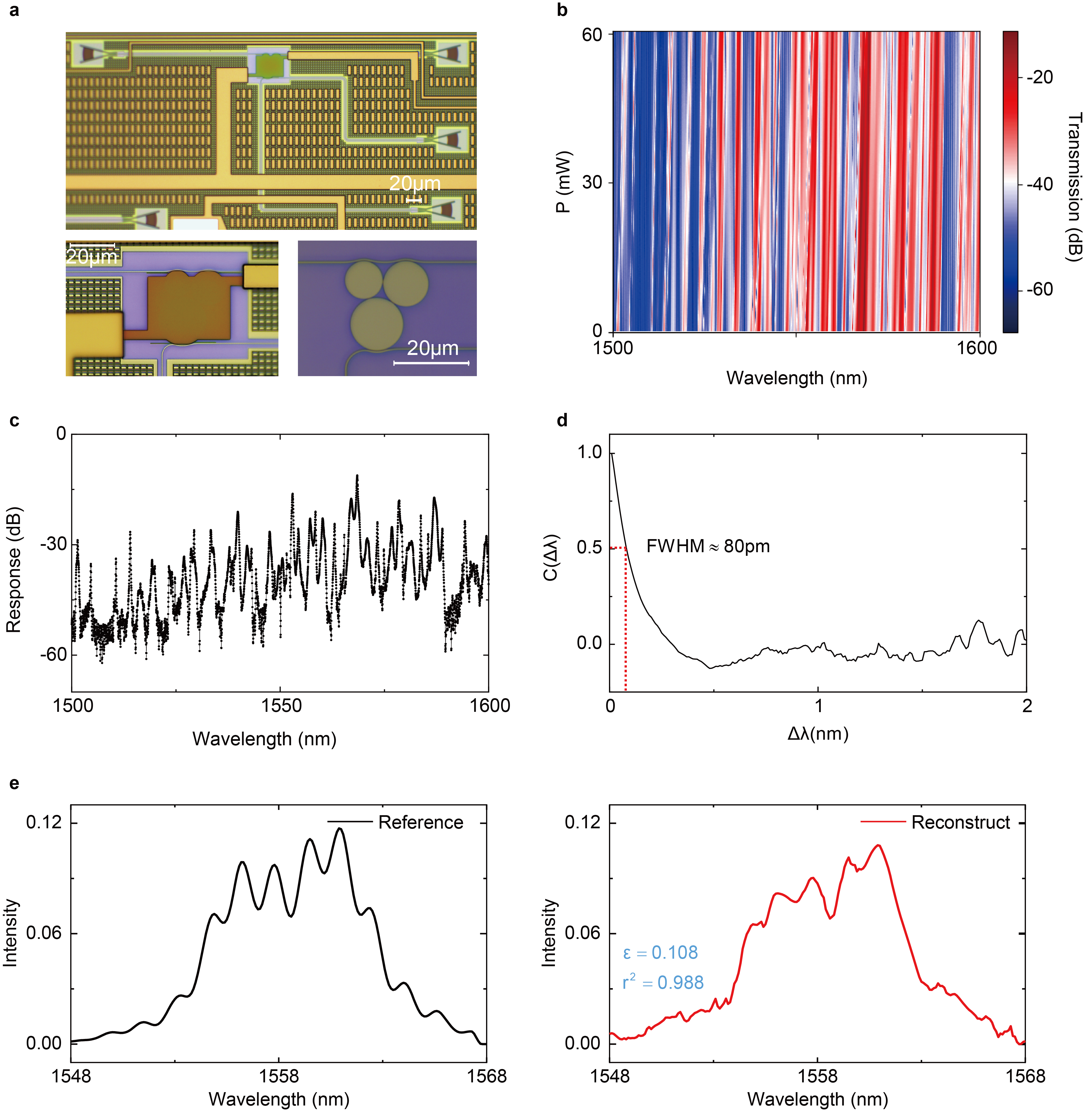


**Fig. S18 | Triatomic PM spectrometer.** **a,** Optical microscopy photo of fabricated triatomic PM spectrometer. **b,** Experimentally pre-calibrated response matrix under heating powers. **c,** Initial transmission spectrum under zero heating power. **d,** Estimated resolution calculation. **e,** Continuous signals reconstruction with *ε* and *r*^2^ labeled. Left: measured by OSA. Right: Reconstruction by PM spectrometer.

**S18. Stability test**

To further validate this stability, we have measured the transmission of our PM spectrometer before and after 5, 10 operations, and after a 14-day period, as shown in Fig. **S19**. The mean squared errors (MSEs) between transmissions after 5 operations, 10 operations, and 14 days compared to the initial measurement are 2.8781×10^–10^, 9.9690×10^–10^, and 1.5367×10^–8^, respectively. These results confirm that the transmission remains consistent with the initial measurement, indicating that mechanical aspects such as coupling gaps maintain their integrity across multiple operations.


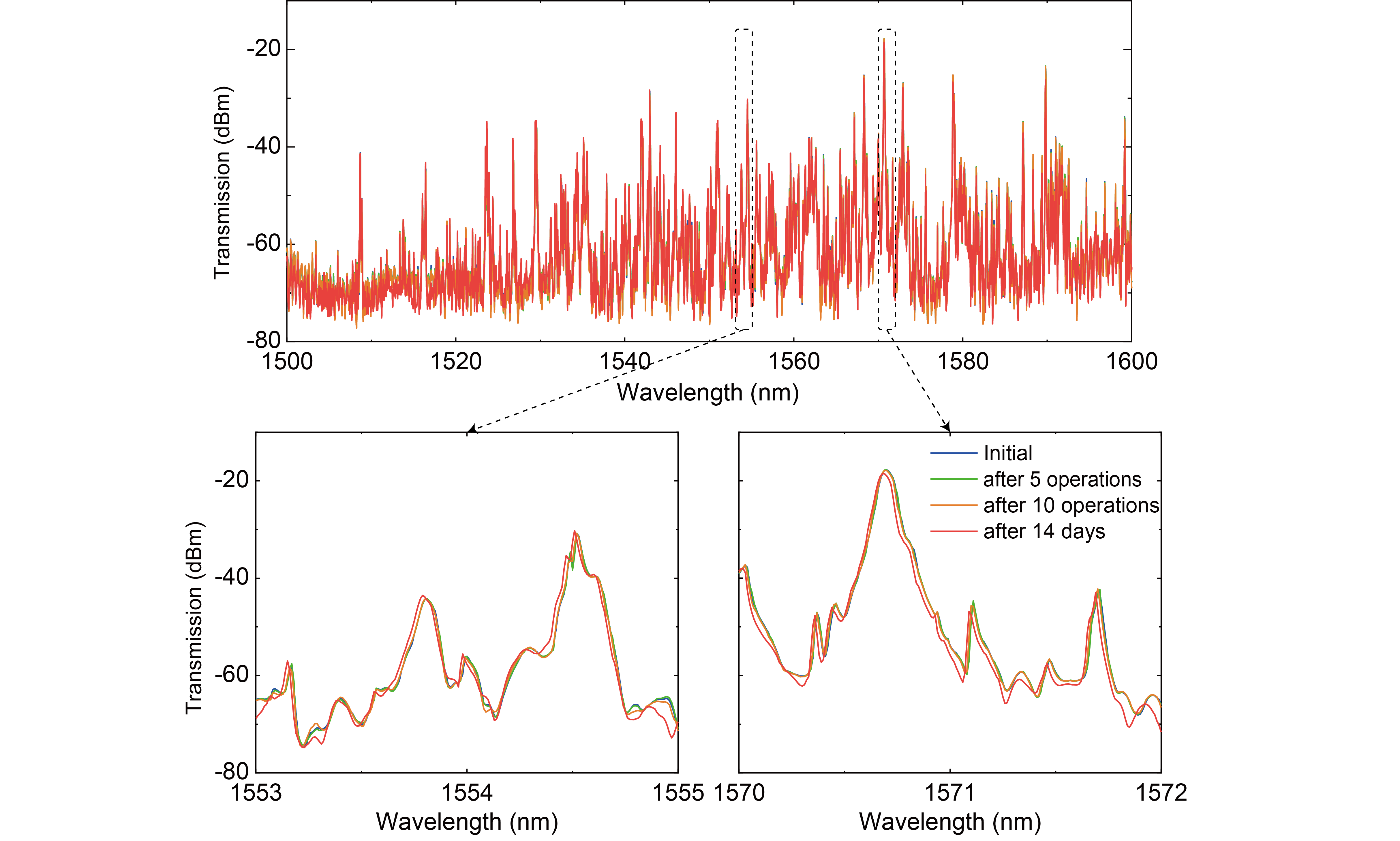


**Fig. S19 |** **Stability test.** Transmission spectra measured initially, after 5 operation cycles, after 10 operation cycles and after 14 days.

Additionally, we performed reconstruction measurements of a single peak signal after 5 operations, 10 operations, and 14 days, as depicted in Fig. **S20**. Despite slight picometer-scale shifts in the resolved wavelength after 10 operations and 14 days—likely due to temperature fluctuations—the impact remains minimal for most applications. Notably, in our design of an ultra-compact, CMOS-compatible spectrometer that prioritizes cost-effectiveness and energy efficiency, we opted not to include a temperature controller. The minor discrepancies between initial and subsequent transmissions and peak reconstructions can be attributed to ambient temperature variations in the lab. These can be compensated for with a temperature controller or through recalibration of the response matrix under different ambient conditions.


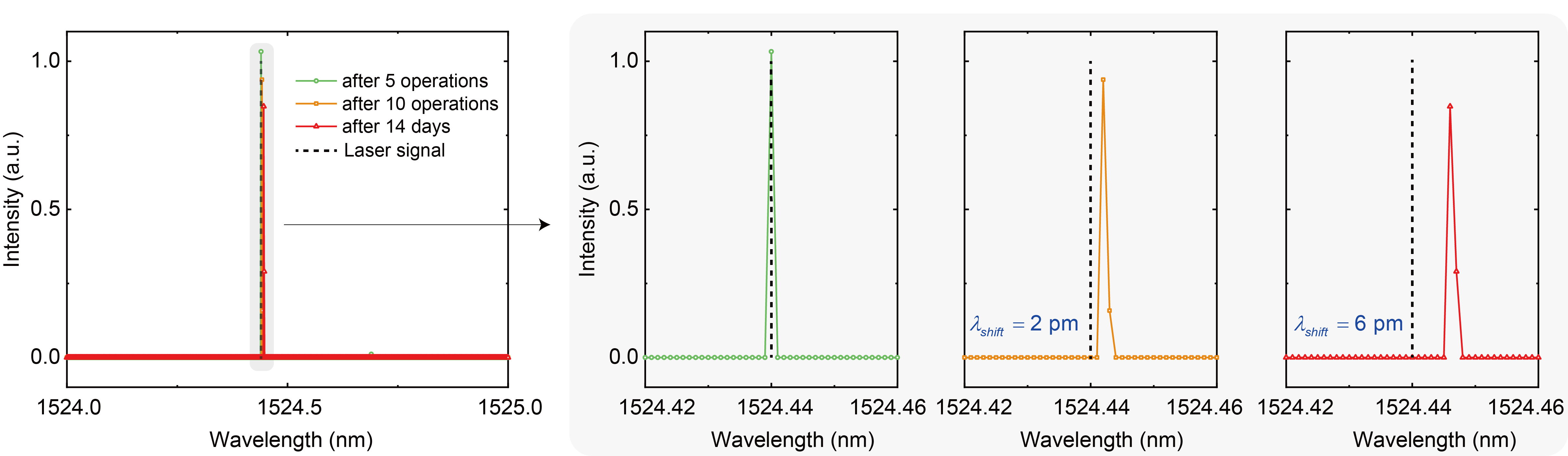


**Fig. S20 |** **Stability test.** Reconstruction of a single-peak signal after 5 operations, 10 operations, and after 14 days.

**S19. Sensitivity**

We have conducted experiments to explore the sensitivity limitation of the PM spectrometer. An optical tunable attenuator is connected after the CW laser source to generate single peak signals with intensities of –20 dBm, –30 dBm, and –40 dBm, respectively. The resolved spectra are exhibited in Fig. **S21** with red lines, and dashed black lines indicating the laser signals. With a noise floor of approximately –40 dBm, at least optical signals with −30 dBm intensity can be resolved, while –40 dBm signals are unresolved, indicating a sensitivity of our PM spectrometer lower than –30 dBm.


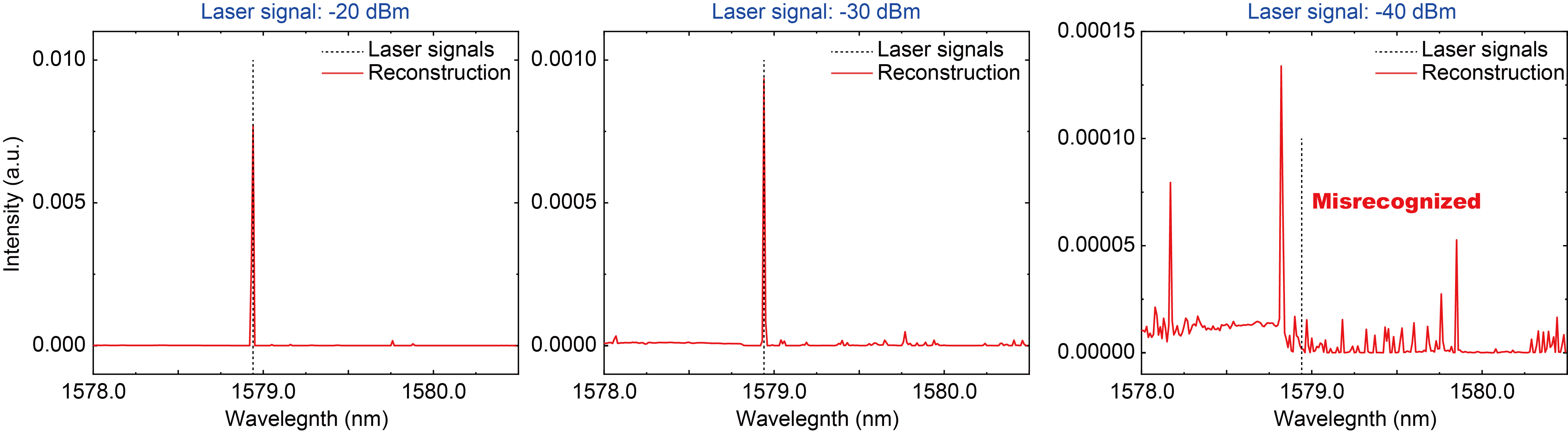


**Fig. S21 |** **Sensitivity test.** Reconstruction results of laser signals with intensity of a, –20 dBm, b, –30 dBm and c, –40 dBm.

**S20. Operational bandwidth extending to other wavelength bands**

The PM spectrometer concept is universal, and the detectable bandwidth is inherently limited by the bandgap of the material used to construct the spectrometer, as well as instrumental bandwidth restrictions. In our case, the operational bandwidth in the PM spectrometer of the configuration we proposed in the manuscript is mainly limited by the bandwidth of grating couplers to achieve into- and off-chip light couplings. Grating couplers are wavelength-sensitive optics that deliver a 3-dB bandwidth of about 39.4 nm in 1550 nm. The operating bandwidth can be easily extended to at least 200 nm by replacing the grating couplers with edge couplers (which is inherently broadband).

Due to its large transparent window, CMOS-compatible fabrication, and low cost, Silicon outstands as the first preferred material suitable for spectroscopy system. Since the TiN heater is embedded in the silicon dioxide, we must consider the overlapping transparent window of silicon and silicon dioxide. Typically, SOI devices should operate within the bandwidth from 1.2 μm to 4 μm. Furthermore, SOI technology optimal performance bandwidth aligns with the Telecommunication band (O-bands to U-band, 1260 nm to 1675 nm). Firstly, silicon exhibits low optical absorption at these wavelengths; secondly, a high refractive index contrast between the silicon core and the insulating silica layer ensures efficient confinement of optical mode; Thirdly, the 1310 nm and 1550 nm wavelengths are especially standard in optical fiber communications. At these wavelengths, optical fibers exhibit minimal attenuation and dispersion, making them ideal for long-distance communication. SOI technology can integrate with these optical fibers efficiently, enabling seamless signal transmission and processing; At last, the 1310 nm and 1550 nm bands are supported by well-established technology and components, including lasers, detectors, and modulators, allowing SOI-based devices to leverage these mature technologies and standards for easier integration and adoption.

Unfortunately, it is currently impossible to provide a generalized equation for directly calculating each dimension parameter when transitioning to other light bands. This is because even the calculation of the effective refractive index of a single-mode waveguide involves numerous equations, assumptions, and perturbation theory, which necessitates numerical calculation with professional software. We can provide the overall process for this design operated in other light bands. First, the width and thickness of the single-mode bus waveguide should be determined based on the operational wavelength. FDE solver in Lumerical software is recommended to verify the cut-off for cut-off for higher-order modes. Subsequently, the FDE solver can be utilized to calculate the radiation loss of bending waveguides to determine the minimum PA radius with acceptable loss, according to operational wavelength. Then, one can select PA radii randomly. The next step involves the FDTD solution in Lumerical software to design the coupling gaps and lengths in order to obtain the desired coupling strengths between PAs and between PAs and bus waveguides. Coupling mode theory can provide an approximate calculation for the coupling length as $L=\frac{\lambda}{\pi\Delta n}\sin^{-1}\sqrt{\mu}$, where Δ*n* is the refractive index difference between symmetric and anti-symmetric modes of the directional coupler structure in the coupling region, and *μ* is the desired coupling strength. Please note, the coupling strength primarily influences the spectral resolution. Finally, perform simulations using the FDTD solution to evaluate the entire structure and tune parameters as needed. The outline of the process for selecting dimension parameters when operated in other light bands, as illustrated in Fig. **S22.**


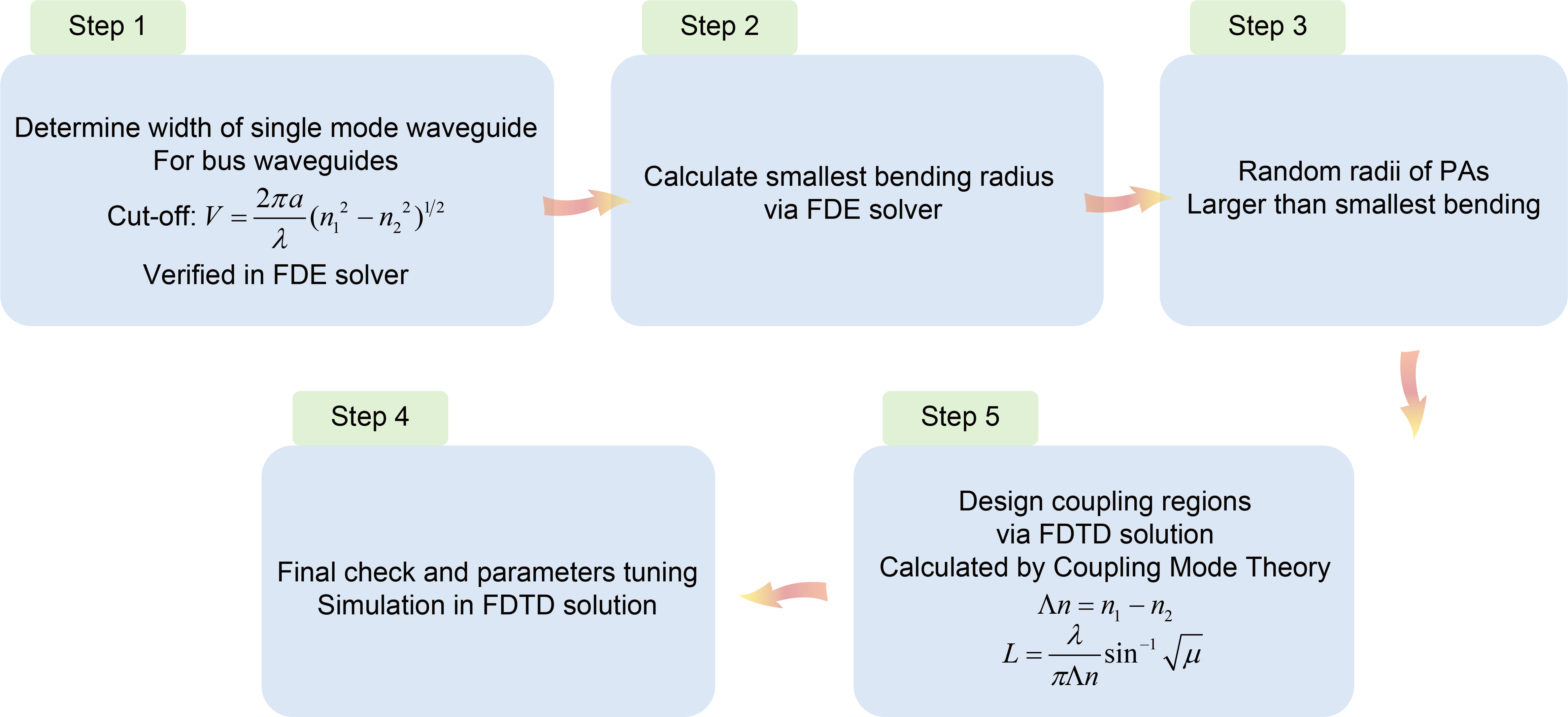


**Fig. S22 | Design flow for other light bands.**

Here, we give designs working in 1.1 μm and 2.0 μm. The radii of microdisk atoms, the width of waveguides, and coupling gaps are adjusted in accordance with wavelength. The simulated transmission spectra with random and disordered behavior and calculated auto-correlation functions are provided in Fig. **S23**, which indicate an effective capability of quasi-random response matrix.


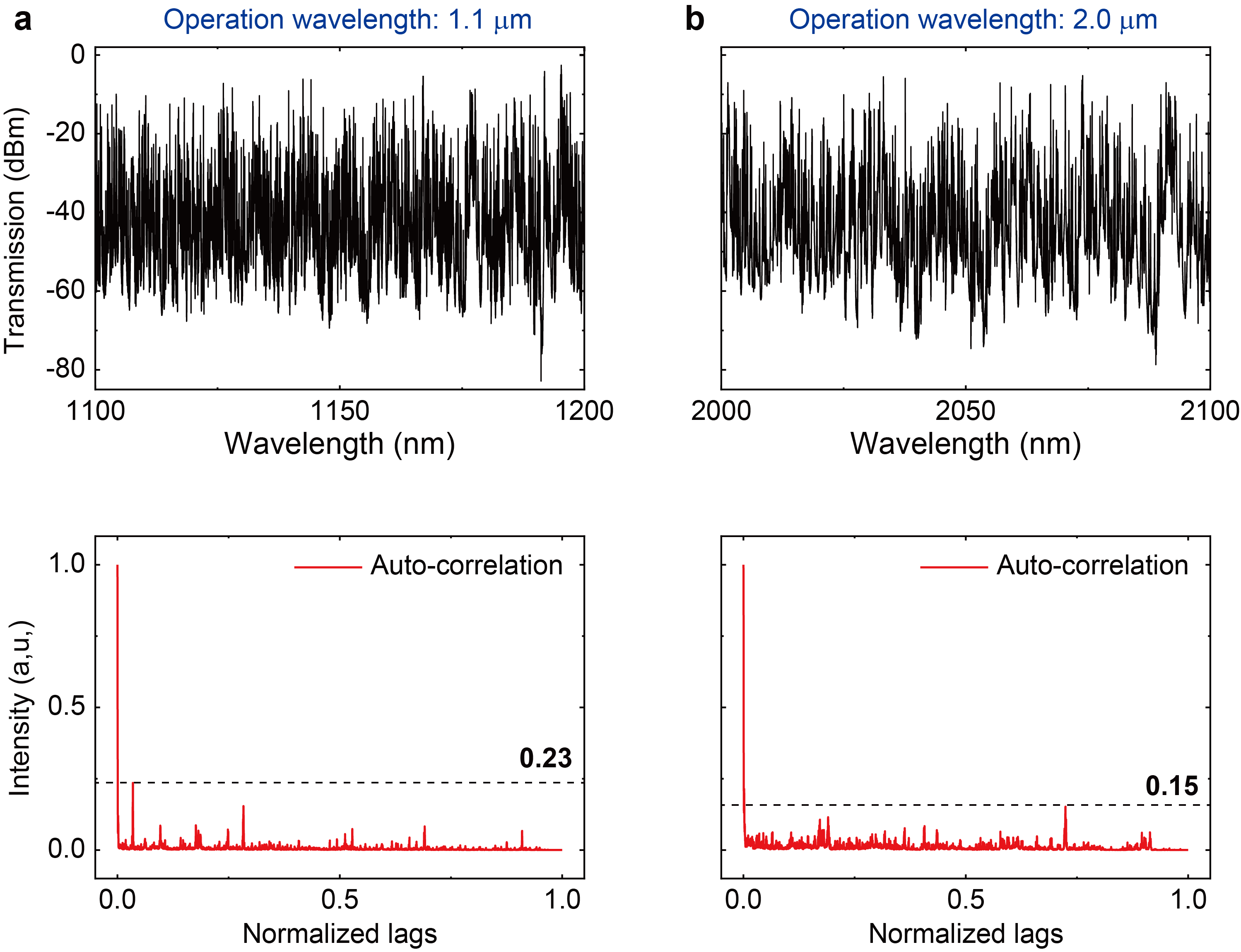


**Fig. S23 |** **Simulated transmission and calculated auto-correlation functions for PM spectrometer designed working in a, 1.1 μm and b, 2.0 μm.**

**S21. Performance comparison for reported computational spectrometers**

| **Method** | **Footprint**  **(**$\boldsymbol{\mu m}^{\boldsymbol{2}}$**)** | **Spatial channels** | **Resolution**  **(nm)** | **Bandwidth (nm)** | **BRR** | **RFP**  **(nm·**$\boldsymbol{\mu m}^{\boldsymbol{2}}$**)** |
| --- | --- | --- | --- | --- | --- | --- |
| Nanowire^3^ | 0.5×75 | 1 | 10 | 130 | 13 | 375 |
| Nanowire^4^ | 0.5×50 | 1 | 5 | 130 | 26 | 125 |
| Scattering multiplexer^5^ | 9.4×14.4 | 10 | 50 | 500 | 10 | 6768 |
| Random scattering medium^6^ | 30×12.8 | 8 | 0.25 | 30 | 120 | 96 |
| stratified waveguide filters^7^ | 35×260 | 32 | 0.45 | 180 | 400 | 4095 |
| black phosphorus photodetector^8^ | 9×16 | 1 | 420 | $7\times{10}^{3}$ | 16.67 | 6.048×10^4^ |
| Random scattering medium^9^ | 50×25 | 25 | 0.75 | 25 | 33.33 | 937.5 |
| Multimode spiral waveguide^10^ | 500×500 | 40 | 0.01 | 2 | 200 | 2500 |
| Random scattering medium^11^ | 200×100 | 13 | 0.3 | 15 | 50 | 6000 |
| Random scattering medium^12^ | 200×100 | 16 | 0.03 | 15 | 500 | 600 |
| Nanobeam Array^13^ | 114×6 | 38 | 5 | 70 | 14 | 3420 |
| Multimode cavities^14^ | 1500×1000 | 1 | 0.005 | 100 | 2×10^4^ | 7500 |
| MZI+MRR^15^ | 520×220 | 64 | 0.02 | 12 | 600 | 2288 |
| Cascaded nanobeam^16^ | 18×18 | 3 | 0.32 | 16 | 50 | 103.68 |
| Single microdisk^17^ | 200×200 | 1 | 0.2 | 20 | 100 | 8000 |
| Cavity-enhanced^18^ | 3.5×10^5^ | 11 | 0.005 | 10 | 2000 | 1750 |
| Coupled MRR^19^ | 60×60 | 1 | 0.04 | 100 | 2500 | 144 |
| DC integrated MRR^20^ | 20×35 | 1 | 0.08 | 100 | 1250 | 56 |
| MZI+MRR^21^ | ~8400×2200 | 4 | 0.03 | 115 | 3833.3 | 1.248×10^5^ |
| Engineered MZI^22^ | 1900×3700 | 1 | 0.01 | 200 | 2×10^4^ | 7.03×10^4^ |
| MRR^23^ | 16×16 | 1 | 0.4 | 10 | 25 | 102.4 |
| Stationary-wave integrated FTS^24^ | 22×512 | 1 | 4 | 96 | 24 | 4.506×10^4^ |
| Spatial heterodyne FTS^25^ | 1.2×10^7^ | 32 | 0.04 | 0.75 | 18.75 | 4.8×10^5^ |
| Digital FTS^26^ | NA | 64 | 0.2 | 20 | 100 | NA |
| Tunable FTS^27^ | 1×10^6^ | 1 | 3 | 65 | 21.67 | 3.00×10^6^ |
| Stationary-wave integrated FTS^28^ | 1×10^7^ | 1 | 5.5 | 500 | 90.91 | 5.5×10^7^ |
| Tunable FTS+MRR^29^ | ~900×240 | 1 | 0.47 | 90 | 191.49 | 1.015×10^5^ |
| Stationary-wave integrated FTS^30^ | 267 × 214 | 1 | 1.2 | 325 | 270.83 | 320.4 |
| Spatial heterodyne FTS^31^ | 5500×6000 | 128 | 0.25 | 200 | 800 | 8.25×10^6^ |
| FTS^32^ | NA | 6 | 0.16 | 180 | 1125 | NA |
| **Our work** | **70×50** | **1** | **0.008** | **100** | **12500** | **28** |

*BRR: Bandwidth to resolution ratio

*RFP: Resolution and footprint product

*MZI: Mach-Zehnder interferometer

*MRR: Microring resonator

*FTS: Fourier-transform spectrometer

*NA: Not applied

Reference

1 Boriskina, S. V. Theoretical prediction of a dramatic Q-factor enhancement and degeneracy removal of whispering gallery modes in symmetrical photonic molecules. *Opt Lett* **31**, 338-340 (2006).

2 Hansen, P. C. *Discrete inverse problems: insight and algorithms*. (SIAM, 2010).

3 Yang, Z. *et al.* Single-nanowire spectrometers. *Science* **365**, 1017-1020 (2019).

4 Zheng, B. *et al.* On‐chip measurement of photoluminescence with high sensitivity monolithic spectrometer. *Advanced Optical Materials* **8**, 2000191 (2020).

5 Liu, T. & Fiore, A. Designing open channels in random scattering media for on-chip spectrometers. *Optica* **7** (2020).

6 Hadibrata, W., Noh, H., Wei, H., Krishnaswamy, S. & Aydin, K. Compact, High‐resolution Inverse‐Designed On‐Chip Spectrometer Based on Tailored Disorder Modes. *Laser & Photonics Reviews* **15** (2021).

7 Li, A. & Fainman, Y. On-chip spectrometers using stratified waveguide filters. *Nat Commun* **12**, 2704 (2021).

8 Yuan, S., Naveh, D., Watanabe, K., Taniguchi, T. & Xia, F. A wavelength-scale black phosphorus spectrometer. *Nature Photonics* **15**, 601-607 (2021).

9 Redding, B., Liew, S. F., Sarma, R. & Cao, H. Compact spectrometer based on a disordered photonic chip. *Nature Photonics* **7**, 746-751 (2013).

10 Redding, B., Fatt Liew, S., Bromberg, Y., Sarma, R. & Cao, H. Evanescently coupled multimode spiral spectrometer. *Optica* **3** (2016).

11 Hartmann, W. *et al.* Waveguide‐Integrated Broadband Spectrometer Based on Tailored Disorder. *Advanced Optical Materials* **8** (2020).

12 Hartmann, W. *et al.* Broadband Spectrometer with Single-Photon Sensitivity Exploiting Tailored Disorder. *Nano Lett* **20**, 2625-2631 (2020).

13 Cheng, Z. *et al.* Generalized Modular Spectrometers Combining a Compact Nanobeam Microcavity and Computational Reconstruction. *ACS Photonics* (2021).

14 Xu, H., Qin, Y., Hu, G. & Tsang, H. K. Cavity-enhanced scalable integrated temporal random-speckle spectrometry. *Optica* **10**, 1177-1188 (2023).

15 Zhang, Z. *et al.* Compact High Resolution Speckle Spectrometer by Using Linear Coherent Integrated Network on Silicon Nitride Platform at 776 nm. *Laser & Photonics Reviews* **15** (2021).

16 Zhang, J., Cheng, Z., Dong, J. & Zhang, X. Cascaded nanobeam spectrometer with high resolution and scalability. *Optica* **9** (2022).

17 Sun, C. *et al.* Scalable On‐Chip Microdisk Resonator Spectrometer. *Laser & Photonics Reviews* **17** (2023).

18 Zhang, L. *et al.* Ultrahigh-resolution on-chip spectrometer with silicon photonic resonators. *Opto-Electronic Advances* **5**, 210100-210100 (2022).

19 Xu, H., Qin, Y., Hu, G. & Tsang, H. K. Breaking the resolution-bandwidth limit of chip-scale spectrometry by harnessing a dispersion-engineered photonic molecule. *Light Sci Appl* **12**, 64 (2023).

20 Xu, H., Qin, Y., Hu, G. & Tsang, H. K. Integrated Single-Resonator Spectrometer beyond the Free-Spectral-Range Limit. *ACS Photonics* **10**, 654-666 (2023).

21 Yao, C. *et al.* Broadband picometer-scale resolution on-chip spectrometer with reconfigurable photonics. *Light Sci Appl* **12**, 156 (2023).

22 Yao, C. *et al.* Integrated reconstructive spectrometer with programmable photonic circuits. *Nature Communications* **14**, 6376 (2023).

23 Zhao, Y. *et al.* Miniaturized computational spectrometer based on two-photon absorption. *Optica* **11**, 399-402 (2024).

24 Le Coarer, E. *et al.* Wavelength-scale stationary-wave integrated Fourier-transform spectrometry. *Nature Photonics* **1**, 473-478 (2007).

25 Velasco, A. V. *et al.* High-resolution Fourier-transform spectrometer chip with microphotonic silicon spiral waveguides. *Optics letters* **38**, 706-708 (2013).

26 Kita, D. M. *et al.* High-performance and scalable on-chip digital Fourier transform spectroscopy. *Nat Commun* **9**, 4405 (2018).

27 Souza, M. C., Grieco, A., Frateschi, N. C. & Fainman, Y. Fourier transform spectrometer on silicon with thermo-optic non-linearity and dispersion correction. *Nature communications* **9**, 665 (2018).

28 Pohl, D. *et al.* An integrated broadband spectrometer on thin-film lithium niobate. *Nature Photonics* **14**, 24-29 (2019).

29 Zheng, S. N. *et al.* Microring resonator-assisted Fourier transform spectrometer with enhanced resolution and large bandwidth in single chip solution. *Nat Commun* **10**, 2349 (2019).

30 Finco, G. *et al.* Monolithic thin-film lithium niobate broadband spectrometer with one nanometre resolution. *Nature Communications* **15**, 2330 (2024).

31 Xu, H., Qin, Y., Hu, G. & Tsang, H. K. Scalable integrated two-dimensional Fourier-transform spectrometry. *Nature Communications* **15**, 436 (2024).

32 Li, A. & Fainman, Y. Integrated silicon Fourier transform spectrometer with broad bandwidth and ultra‐high resolution. *Laser & Photonics Reviews* **15**, 2000358 (2021).
